# Melt inclusion constraints on petrogenesis of the 2014–2015 Holuhraun eruption, Iceland

**DOI:** 10.1007/s00410-017-1435-0

**Published:** 2018-01-12

**Authors:** Margaret E. Hartley, Enikö Bali, John Maclennan, David A. Neave, Sæmundur A. Halldórsson

**Affiliations:** 1 School of Earth and Environmental Sciences, University of Manchester, Manchester, UK; 2 Institute of Earth Sciences, University of Iceland, Reykjavík, Iceland; 3 Department of Earth Sciences, University of Cambridge, Cambridge, UK; 4 Institut für Mineralogie, Leibniz Universität Hannover, Hannover, Germany

**Keywords:** Iceland, Holuhraun, Melt inclusions, Crystallization, Melt barometry

## Abstract

The 2014–2015 Holuhraun eruption, on the Bárðarbunga volcanic system in central Iceland, was one of the best-monitored basaltic fissure eruptions that has ever occurred, and presents a unique opportunity to link petrological and geochemical data with geophysical observations during a major rifting episode. We present major and trace element analyses of melt inclusions and matrix glasses from a suite of ten samples collected over the course of the Holuhraun eruption. The diversity of trace element ratios such as La/Yb in Holuhraun melt inclusions reveals that the magma evolved via concurrent mixing and crystallization of diverse primary melts in the mid-crust. Using olivine–plagioclase–augite–melt (OPAM) barometry, we calculate that the Holuhraun carrier melt equilibrated at 2.1 ± 0.7 kbar (7.5 ± 2.5 km), which is in agreement with the depths of earthquakes (6 ± 1 km) between Bárðarbunga central volcano and the eruption site in the days preceding eruption onset. Using the same approach, melt inclusions equilibrated at pressures between 0.5 and 8.0 kbar, with the most probable pressure being 3.2 kbar. Diffusion chronometry reveals minimum residence timescales of 1–12 days for melt inclusion-bearing macrocrysts in the Holuhraun carrier melt. By combining timescales of diffusive dehydration of melt inclusions with the calculated pressure of H_2_O saturation for the Holuhraun magma, we calculate indicative magma ascent rates of 0.12–0.29 m s^−1^. Our petrological and geochemical data are consistent with lateral magma transport from Bárðarbunga volcano to the eruption site in a shallow- to mid-crustal dyke, as has been suggested on the basis of seismic and geodetic datasets. This result is a significant step forward in reconciling petrological and geophysical interpretations of magma transport during volcano-tectonic episodes, and provides a critical framework for the interpretation of premonitory seismic and geodetic data in volcanically active regions.

**Electronic supplementary material:**

The online version of this article (10.1007/s00410-017-1435-0) contains supplementary material, which is available to authorized users.

## Introduction

The recent eruption at Holuhraun within the Bárðarbunga volcanic system in central Iceland began on 29 August 2014 and, with the exception of a short break in activity on 30 August, continued until 27 February 2015, producing over 1.4 km^3^ of dominantly plagioclase–phyric lava and tephra. The event was one of the best-monitored basaltic fissure eruptions that have ever occurred, and presents a unique opportunity to link petrological and geochemical data with geophysical observations during a major rifting episode. It also provides an excellent opportunity to investigate mechanisms of melt generation and its subsequent storage and transport within Iceland’s axial rift zones.

We present a detailed petrological and geochemical characterization of erupted products from Holuhraun. We use these data to evaluate pressures of melt mixing, storage, and equilibration in the Holuhraun magmatic system. We present a new implementation of the olivine–plagioclase–augite–melt (OPAM) barometer (Yang et al. 1996) to evaluate melt equilibration pressures, whereby melt compositions that are not multiply saturated can be identified and discarded, and the systematic error in returned pressure estimates is minimised. We use diffusion chronometry to estimate minimum residence times of macrocryst phases in the Holuhraun melt, and obtain indicative ascent rates for the Holuhraun magma. Finally, we compare our petrological observations with geophysical data to evaluate the fidelity of petrological and geochemical datasets in recovering the apparent magma storage and transport during a major volcano-tectonic episode. In studies of previous, similar volcano-tectonic events in Iceland, petrologists have typically evaluated the depths of magma storage and transport as being significantly deeper than the recorded seismicity. Here, for the first time, we have recovered petrological constraints on melt storage and transport pressures that are in very good agreement with seismic and geodetic determinations from the same event. This marks a significant step forward towards the reconciliation of petrological and geophysical datasets. Our methods and results are applicable to the interpretation of melt transport pathways during historical eruptions where geophysical and geodetic data are not available, and may lead to significant improvements in the forecasting of future fissure eruptions, both in Iceland and further afield.

## Geological background

### Melt transport in Icelandic systems

Volcano-tectonic episodes in Iceland include the 1975–1984 Krafla Fires (Björnsson et al. 1977; Einarsson and Brandsdóttir 1979), Askja 1874–1876 (Macdonald et al. 1987; Hartley and Thordarson 2013), Laki 1783–1784 and Eldgja 934 (Sigurdsson and Sparks 1978; Thordarson and Self 1993). With the exception of the Krafla Fires, no high-resolution seismic or geodetic data exist for these volcano-tectonic episodes. Thus, evaluation of magma supply and transport for Icelandic historic volcano-tectonic episodes has, to date, relied on the interpretation of petrological and geochemical data, supported by low-resolution records of seismicity and/or ground deformation gleaned from historical accounts when available (e.g., Hartley and Thordarson 2012). This has resulted in two principal end-member hypotheses describing the possible nature of magmatic plumbing systems feeding fissure eruptions in volcanic rift zones. First, replenishment and pressurization of a magma chamber beneath the volcanic edifice may result in lateral magma injection into the mid to upper crust in the direction of maximum tensile stress, resulting in laterally propagating seismicity and potentially a fissure eruption along the rift zone (e.g., Sigurdsson and Sparks 1978). Magma withdrawal from a shallow magma chamber may also result in caldera subsidence at the volcanic edifice (e.g., Stix and Kobayashi 2008, and references therein). Alternatively, fissure eruptions along rift zones may be driven by pressurization of lower crustal magma reservoirs and the injection of subvertical dykes towards the surface, with associated movement on caldera ring faults resulting from the concentration of dyke-induced stress fields around pre-existing faults and/or shallow magma chambers (e.g., Gudmundsson 1995; Gudmundsson et al. 2014). These models have been discussed extensively with respect to volcano-tectonic episodes for volcanic edifices including Kilauea, Hawaii (Ryan 1988; Baker and Amelung 2012); the Afar region of Ethiopia (Wright et al. 2006; Jeir et al. 2009; Ebinger et al. 2010); and mid-ocean ridge segments (e.g., Fialko and Rubin 1998; Dziak et al. 1995; Escartín et al. 2014).

**Fig. 1:**
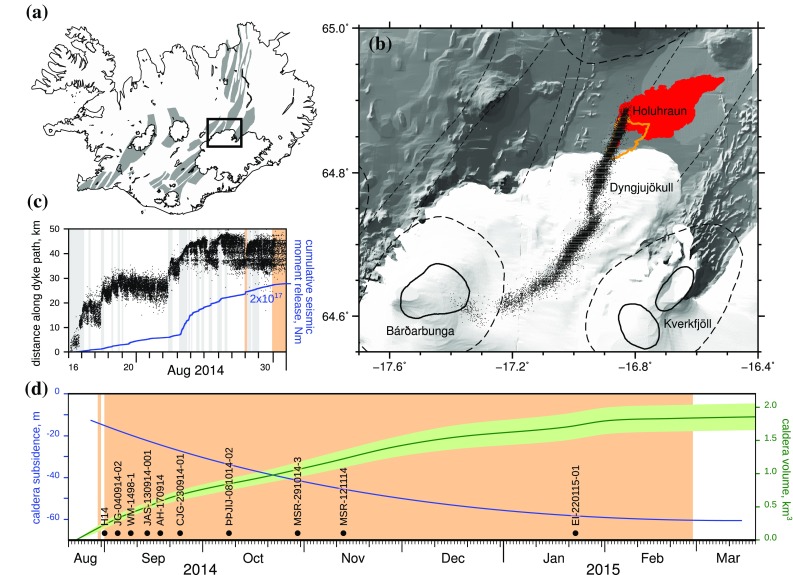
**a** Map of Iceland showing the neovolcanic rift zones. **b** Map of central Iceland showing central volcanoes and their fissure swarms. The 2014–2015 Holuhraun lava is shaded in red; the orange line shows the margins of older Holuhraun lavas. Black points show epicentres of earthquakes in the dyke extending between Bárðarbunga central volcano and the Holuhraun eruption site (data from Ágústsdóttir et al. 2016). **c** Propagation of pre-eruptive seismicity between Bárðarbunga and the eruption site, modified after Ágústsdóttir et al. (2016). Dyke propagation phases are shaded in grey, and eruption periods shaded in orange. **d** Subsidence in the centre of Bárðarbunga caldera and the evolution of the subsidence volume, modified after Gudmundsson et al. (2016). Black circles indicate samples used in this study and their date of eruption

The 2014–2015 event at Holuhraun is the best-monitored Icelandic volcano-tectonic episode ever to have occurred. For the first time, we have interlinked high-resolution datasets describing the seismicity (Sigmundsson et al. 2015; Ágústsdóttir et al. 2016) and ground deformation (Gudmundsson et al. 2016), as well as changes in volcanic plume chemistry (Gauthier et al. 2016), all of which can be directly linked to the petrology and geochemistry of erupted products collected at regular intervals over the course of the eruption (Fig. 1). The Holuhraun eruption thus represents a unique opportunity to integrate the petrological characteristics of the erupted products from a volcano-tectonic episode with various geophysical datasets, and hence to evaluate the most likely model of melt transport in the light of multiple independent, high-quality datasets. This not only enhances our ability to interpret magma storage and transport pathways during historic volcano-tectonic episodes, where such geophysical data are unavailable, but also provides a vital framework for the interpretation of premonitory seismic and geodetic data in volcanically active regions, which is crucial to improving volcano monitoring and the response to future eruptive hazards.

### Geophysical observations of the Holuhraun volcano-tectonic episode

Intense seismicity on the Bárðarbunga volcanic system commenced on 16 August 2014, with the distribution of seismicity consistent with a radial dyke propagating northeastwards from Bárðarbunga volcano. Simultaneously, GPS observations recorded subsidence at Bárðarbunga caldera, and extension perpendicular to the propagating dyke (Sigmundsson et al. 2015). Dyke propagation continued at a variable rate until 29 August 2015, with earthquake hypocentral depths being tightly concentrated between 5 and 7 km along the entire 48 km dyke path (Ágústsdóttir et al. 2016). On 29 August, a fissure eruption commenced at the site of vents previously active during two older eruptions dated at 1797 and c. 1862–1864 AD (Hartley and Thordarson 2013). Following a period of 2–3 weeks of intense activity with magma discharge rates up to 8 × 10^5^ kg s^−1^ (Gíslason et al. 2015), both the lava mass flow rate at Holuhraun and the rate of caldera subsidence at Bárðarbunga decreased exponentially until the eruption ended after 181 days of activity (Gudmundsson et al. 2016). The total volume of caldera subsidence, 1.8 ± 0.2 km^3^, is comparable with a combination of the intruded magma volume (0.5 ± 0.1 km^3^) and the total erupted magma output from the Holuhraun vents (~ 1.44 km^3^; Pedersen et al. 2017). Three small ice cauldrons formed along the dyke path probably indicate the sites of small subglacial eruptions (Reynolds et al. 2017), which are not included in this erupted volume estimate. Both seismic and geodetic observations are consistent with the magma erupted at Holuhraun being fed by a lateral dyke extending over 45 km from Bárðarbunga central volcano (Sigmundsson et al. 2015; Ágústsdóttir et al. 2016; Gudmundsson et al. 2016). Here, we interrogate the link between Bárðarbunga and Holuhraun using an extensive melt inclusion dataset.

## Samples and analytical methods

Fresh, glassy samples of magmatic tephra were collected at regular intervals during the Holuhraun eruption. The ten samples selected for this study span the entire course of the eruption (Fig. 1; Table 1). Nine samples consist of proximal tephra fall collected near the main eruptive vent, and are known to have erupted within 2 days of the date of collection. The tenth sample consists of glassy scoria clasts from the flanks of Baugur that were likely deposited up to 2 weeks prior to the date of collection.

**Table 1:** Tephra samples from the 2014–2015 Holuhraun eruption analysed in this study

Sample	Date of collection	Host macrocrysts	Melt inclusions	Embayments	Matrix glasses
H14	31 Aug 2014	plg, olv	39 (+ 5)	1 (+ 1)	3 (+ 2)
JG-040914-02	04 Sep 2014	plg	3		1
WM-1498-1	08 Sep 2014	plg	8		1
JAS-130914-001	13 Sep 2014	plg, olv, cpx	13 (+ 3)	1	1
AH-170914	17 Sep 2014	plg	3		2
CJG-230914-01	23 Sep 2015	plg	6		
ÞÞJIJ-081014-02	08 Oct 2014	plg	8		1
MSR-291014-3	20 Oct 2014	plg			2
MSR-121114	12 Nov 2014	plg	4		1
EI-220115-01	22 Jan 2015	plg, olv	15		1
Total			99 (+ 8)	2 (+ 1)	13 (+ 2)

For each sample, the melt inclusion-bearing host macrocryst phases are indicated alongside the number of melt inclusions, embayments and matrix glasses analysed by SIMS. Numbers in parentheses show repeat analyses used to assess melt inclusion homogeneity and monitor analytical reproducibility

Plagioclase, olivine and clinopyroxene macrocrysts in the size range 250–3000 μm were hand-picked from crushed tephra samples, mounted in epoxy resin, and polished to expose glassy melt inclusions at the surface.

Volatile (H_2_O, F, and Cl), light lithophile, trace and rare-earth elements were analysed in 99 melt inclusion glasses (88 hosted in plagioclase, 7 in olivine, and 4 in clinopyroxene), 2 glassy embayments, and 13 matrix glasses (Table 1) by secondary ion mass spectrometry (SIMS) using the Cameca ims-4f instrument at the University of Edinburgh. CO_2_ was also measured prior to performing the trace element analyses. Precision and accuracy for the trace element analyses were monitored by repeat analyses of standards NIST-SRM610, BCR-2G, KL2-G, and GSD-1G. 1*σ* accuracy was consistently 10% or better. Precision was generally better than ± 2% for trace elements in high abundance (e.g., La, Sr, and Zr) and ± 10% for trace elements in low abundance (e.g., Yb and Lu). Following SIMS analyses, major, minor, and volatile (S and Cl) elements in the same inclusions were measured by electron microprobe (EPMA) using the Jeol JXA-8230 Superprobe at the University of Iceland. Volatile systematics and degassing behaviour of the Holuhraun magma are discussed in detail by Bali et al. (2018). Full details of analytical methods and compositional data are provided as supplementary material.

### Petrography of melt inclusions

**Fig. 2:**
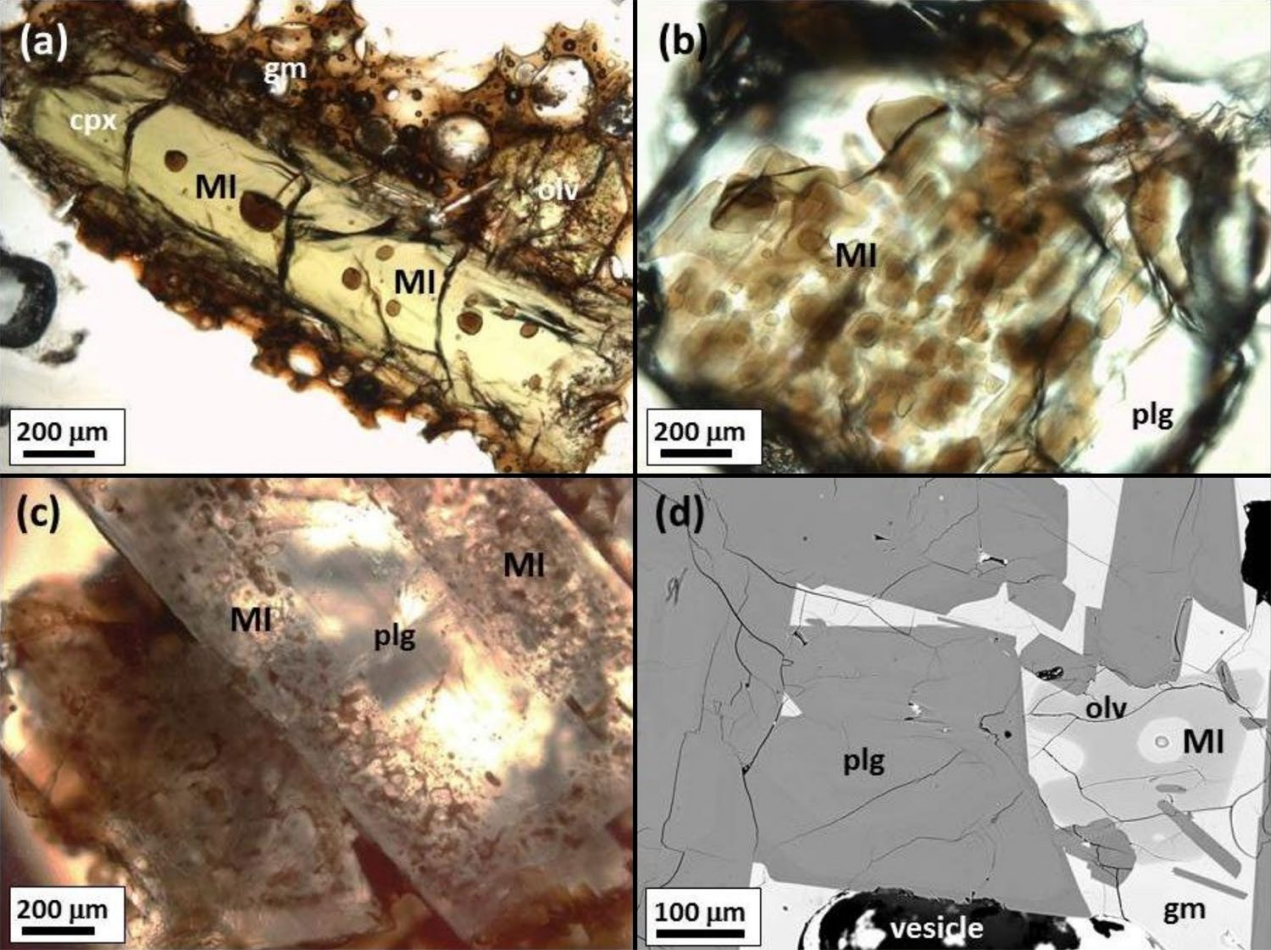
Petrographic characteristics of silicate melt inclusions in macrocrysts and microphenocrysts from the 2014–2015 Holuhraun eruption. **a** Randomly distributed spherical primary melt inclusions (MI) in a clinopyroxene (cpx) macrocryst surrounded by groundmass glass (gm); sample JAS-130914-001. **b** Vermicular primary melt inclusions in a plagioclase (plg) macrocryst; sample MSR-291014-3. **c** Primary melt inclusions in a plagioclase macrocryst rim; sample AH-170914. A trail of pseudosecondary melt inclusions is located along a healed fracture in the macrocryst core. **d** Back-scattered electron image of a primary melt inclusion in an olivine (olv) microphenocryst in sample EI-220115. The circular feature in the melt inclusion is a pit formed during SIMS analysis

Both macrocrysts (here used to describe crystals > 0.5 mm in diameter) and microphenocrysts (< 0.5 mm) from Holuhraun erupted products contain silicate melt inclusions (Fig. 2). The melt inclusions range from 15 to 174 μm in their longest dimension. Most are spherical or elliptical in cross section; a small number have irregular or elongate geometries. Spherical melt inclusions are most commonly situated in macrocryst and microphenocryst cores (Fig. 2a, d) and can be regarded as primary melt inclusions. Irregular-shaped melt inclusions are present in different textural positions. Some are situated in macrocryst cores (Fig. 2b) or within growth zones (Fig. 2c): these are interpreted as texturally primary inclusions, trapped at different stages of mineral growth. Irregular-shaped inclusions are also observed along healed fractures of macrocrysts (Fig. 2c) and are regarded as secondary or pseudosecondary inclusions according to the nomenclature of Roedder (1984). Most secondary and pseudosecondary inclusions are less than 15 µm in diameter, and too small to be analysed by ion microprobe (Fig. S3). It was not possible to determine the genetic relationship between the melt inclusion and host crystal from petrographic observations in some crystal fragments.

## Results

### Major elements

Measured melt inclusion compositions in all host minerals lie between 5.14 and 11.91 wt% MgO (average 8.07 wt. MgO; Fig. S1). However, major element compositions of melt inclusions can be greatly affected by post-entrapment crystallization (PEC) of the host mineral onto the inclusion walls. For olivine-hosted inclusions, PEC causes a reduction in melt inclusion Mg#; for plagioclase-hosted inclusions, Mg# is unaffected, but Al_2_O_3_ and CaO are removed from the inclusion by PEC. Olivine-hosted inclusions were corrected for PEC using Petrolog3 (Danyushevsky and Plechov 2011), following the methodology of Danyushevsky et al. (2000).

There is no universally accepted procedure for correcting PEC in plagioclase-hosted melt inclusions. We therefore employed an empirical correction based on TiO_2_–Al_2_O_3_ systematics, similar to that of Neave et al. (2017). TiO_2_ is incompatible during the crystallization of all phases, whereas Al_2_O_3_ is the most modified component during PEC in plagioclase-hosted inclusions. The unmodified Al_2_O_3_ content of the plagioclase-hosted inclusions can therefore be estimated at a given TiO_2_ content based on the TiO_2_–Al_2_O_3_ relationship shown by Holuhraun and Bárðarbunga (Breddam 2002; Óladóttir et al. 2011; Neave et al. 2014) glass compositions (*R*^2^ = 0.67, *p* ≪ 0.01). Melt inclusions were corrected for PEC by incrementally adding the host plagioclase composition back into the inclusion until its TiO_2_–Al_2_O_3_ systematics were consistent with Icelandic tholeiitic glasses: that is, until Al_2_O_3_ in the inclusion reached the value predicted from TiO_2_ based on a linear regression through the glass data (Figs. S1, S2). Following this procedure, 89% of the plagioclase-hosted inclusions had ${\text{Kd}}_{{{\text{Ab-An}}}}^{{{\text{plg-liq}}}}$ within the range 0.27 ± 0.11 relevant for melt–plagioclase equilibration above 1050 °C (Putirka 2008). Eleven inclusions, one hosted in An_81_ and ten in An_>84_ plagioclases, had ${\text{Kd}}_{{{\text{Ab-An}}}}^{{{\text{plg-liq}}}}$ lower than the equilibrium range. Applying any further PEC correction to these inclusions to comply with the equilibrium criterion resulted in unrealistically high Al_2_O_3_ contents compared with Bárðarbunga and other Icelandic tholeiitic glasses. Similarly unrealistic melt compositions were obtained by Neave et al. (2017) when applying equilibrium-based PEC corrections to high-anorthite plagioclase-hosted inclusions from the nearby Grímsvötn volcanic system.

**Fig. 3:**
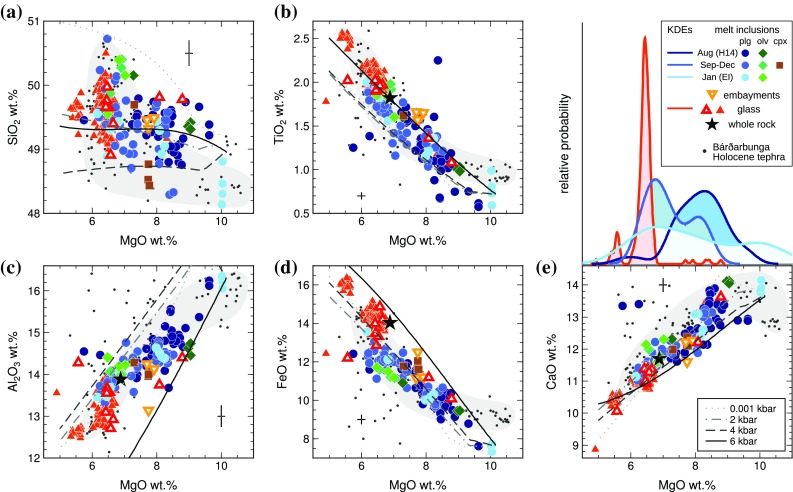
Major elements of melt inclusions, embayments, and glasses from the 2014–2015 Holuhraun eruption. Melt inclusion compositions have been corrected for post-entrapment crystallization. Small red triangles show glass compositions; large open red triangles show the glass samples in which trace elements were analysed. Black stars show the mean whole-rock composition. Grey-shaded fields show typical Holocene tephra glass compositions from the Bárðarbunga volcanic system; small black circles are Bárðarbunga tephra analyses from Thordarson et al. (1998) and Óladóttir et al. (2011). Error bars are 1*σ*. Solid and dashed lines show fractional crystallization models calculated for the mean primitive melt inclusion composition, calculated over a range of pressures using the mineral–melt equilibria of Langmuir et al. (1992). The kernel density estimates above panel **e** show the distribution of measured MgO contents for Holuhraun matrix glasses (red), melt inclusions in samples collected in August 2014, (dark blue), melt inclusions from September–December 2014 (mid blue), and melt inclusions from January 2015 (light blue)

We conducted a further test for equilibrium by comparing the output from a plagioclase–melt thermometer [Eq. 24a of Putirka (2008); standard error of estimate (SEE) ± 36 °C] with an independent melt thermometer [Eq. 16 of Putirka (2008); SEE ± 26 °C]. Both equations require the input of a pressure term. For any given inclusion, varying the input pressure between 1 and 5 kbar produced temperature differences up to 30° in the case of the plagioclase–melt thermometer, and up to 16° for the melt thermometer. Since these differences are within the SEE of the respective thermometers, we chose to assume a uniform input pressure of 3 kbar. The thermometers returned temperatures within ± 20 °C of one another for all but nine plagioclase-hosted inclusions, including the six most primitive inclusions. Additional PEC correction was applied to these inclusions such that the thermometers returned temperatures within ± 20 °C, while taking care not to over-correct the inclusion compositions and violate the TiO_2_–Al_2_O_3_ and MgO–Al_2_O_3_ systematics of Icelandic tholeiitic glasses. After this PEC correction, the six most primitive inclusions have slightly lower FeO, and higher CaO, than the only published primitive glass analyses from the Bárðarbunga volcanic system measured at Kistufell (Breddam 2002) (Fig. 3). However, it was not possible to match simultaneously all the PEC-corrected major element contents to the Kistufell trend, with or without satisfying the requirement for thermal equilibrium.

Clinopyroxene-hosted melt inclusions were corrected for PEC by adding the host composition back into the inclusion until ${\text{Kd}}_{{{\text{Fe-Mg}}}}^{{{\text{cpx-liq}}}}$ fell within the equilibrium range 0.28 ± 0.08 (Putirka 2008), and the predicted DiHd, EnFs, and Jd components based on the corrected melt composition were within 15% of the measured host clinopyroxene composition.

The mean PEC correction across all the melt inclusions was 5%. Ten plagioclase-hosted inclusions required PEC corrections > 10%, with the largest correction being 16%. One inclusion was corrected for 1% post-entrapment dissolution of the host plagioclase back into the melt inclusion. After correction for PEC, the plagioclase-hosted melt inclusions range from 10.04 to 5.75 wt% MgO (Fig. 3). PEC-corrected olivine- and clinopyroxene-hosted melt inclusions and embayments contain between 9.05 and 6.48 wt% MgO. The embayments and PEC-corrected inclusions are compositionally similar to Holocene tephras erupted from the Bárðarbunga volcanic system (Óladóttir et al. 2011) (Fig. 3).

Matrix glass compositions were measured immediately adjacent to large plagioclase macrocrysts in sample H14, and in vesicular tephra glass shards for all other samples. Three glasses measured adjacent to large plagioclases in sample H14 are compositionally heterogeneous, containing 5.57, 8.09, and 8.79 wt% MgO. The vesicular tephra glasses cluster around 6.5 wt% MgO, and are compositionally similar to the most evolved melt inclusions (Fig. 3). They are also compositionally identical to vesicular tephra glass shards produced throughout the Holuhraun eruption (*n* = 172; Gudmundsson et al. 2016). In the following discussion, we assume that the mean vesicular glass composition is representative of the Holuhraun carrier melt. At atmospheric pressure, liquidus temperature of $1170_{{ - 5}}^{{+10}}$ °C (Gudmundsson et al. 2016), and oxygen fugacity of FMQ + 0.2 (Bali et al. 2018), this composition is in equilibrium with Fo_72_ olivine, An_69_ plagioclase, and Mg# = 75 clinopyroxene.

### Trace elements

**Fig. 4:**
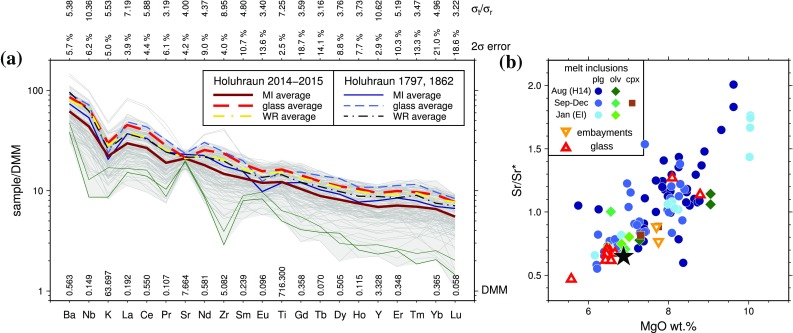
**a** Multi-element diagram for Holuhraun melt inclusions and glasses. Concentrations are normalised to depleted MORB mantle (Workman and Hart 2005) with normalisation values shown along the bottom and 2*σ* errors shown along the top of the plot. The shaded area shows the range of melt inclusion and glass compositions; individual inclusions are shown by the thin grey lines. Variability in melt inclusion compositions is reported as signal-to-noise ratios (*σ*_t_/*σ*_r_) for each element, shown at the top of the plot. Dark green lines show two melt inclusions with moderate depletions in high field strength elements (Zr/Y < 2.1), visible by their negative Zr anomalies. Melt inclusions and glasses from the 2014–2015 eruption have near-identical average compositions to older eruptions at Holuhraun in 1797 and 1862 (data from Hartley and Thordarson 2013). **b** Sr/Sr* vs. MgO in Holuhraun whole-rock (black star), melt inclusions, embayments, and glasses

Trace element compositions in all melt inclusions were corrected for PEC using the partition coefficients collated by O’Neill and Jenner (2012). The PEC-corrected inclusions have very similar average trace element concentrations to the Holuhraun glasses and whole-rock samples, with melt inclusions having slightly lower average trace element abundances than the average glass and whole rock (Fig. 4a). The trace element concentrations measured in melt inclusions, glasses, and whole rocks from the 2014–15 eruption are near-identical to those from the two older eruptions from the Holuhraun vents (Hartley and Thordarson 2013). All trace elements are significantly variable at the 99% confidence level with signal-to-noise ratios *σ*_t_/*σ*_r_ greater than 1.71 (3.22 ≤ *σ*_t_/*σ*_r_ ≤ 10.62), where *σ*_t_ is the true variability within a sample set and *σ*_r_ is an estimate of analytical error (Maclennan et al. 2003).

Strontium anomalies in melt compositions can be quantified by the parameter Sr/Sr* = ${\text{S}}{{\text{r}}_n}/{({\text{C}}{{\text{e}}_n} \times {\text{N}}{{\text{d}}_n})^{0.5}}$, where the subscript *n* indicates chondrite-normalised values. Sr/Sr* in the Holuhraun melt inclusions correlates positively with MgO, which is consistent with crystallization of a plagioclase-bearing assemblage: this is evident in the slight negative Sr anomalies in matrix glasses and some melt inclusions (Fig. 4a) which are indicative of plagioclase crystallization from the melt prior to inclusion trapping. Large positive Sr/Sr* anomalies, accompanied by low La concentrations and low La/Yb, have been suggested to be indicative of dissolution–reaction–mixing (DRM) processes involving plagioclase dissolution into the melt prior to inclusion trapping (e.g., Danyushevsky et al. 2003). Icelandic tholeiites with similar Sr concentrations to the Holuhraun melt inclusions (120–190 ppm Sr) are expected to have Sr/Sr* between 0.5 and ~ 1.9 (Gurenko and Sobolev 2006). Of the 99 Holuhraun inclusions analysed, 59 have Sr/Sr* < 1 and 91 have Sr/Sr* < 1.5 (Fig. 4b). While DRM processes are predicted to produce a negative correlation between La/Yb and Sr/Sr*, there is no correlation between these ratios (*R*^2^ = 0.02) in the Holuhraun inclusions. Furthermore, while the inclusions with Sr/Sr* > 1.5 have low La contents of 2.49–3.52 ppm, they are not systematically associated with low La/Yb: the inclusions with Sr/Sr* > 1.5 have 1.51 < La/Yb < 3.90, compared with 1.43 < La/Yb < 5.14 in the remainder of the melt inclusion suite. These observations indicate that while DRM processes may have had a minor effect on a small number of Holuhraun melt inclusions, it is not the dominant control on trace element variability in these inclusions.

**Fig. 5:**
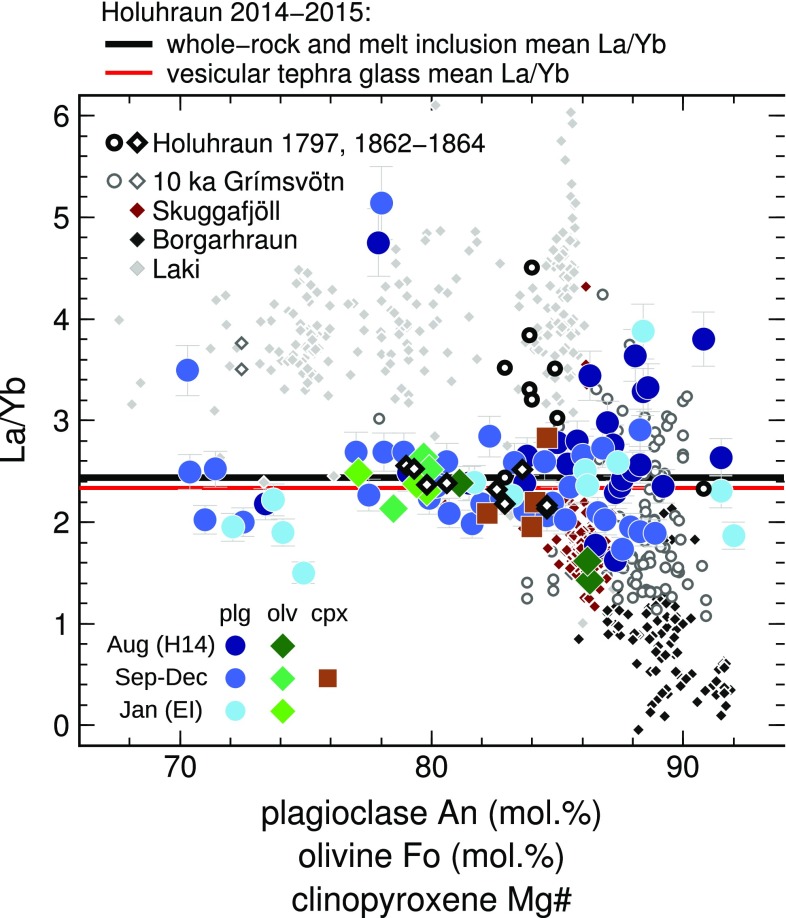
La/Yb vs. host macrocryst composition for Holuhraun melt inclusions. Error bars are 2*σ*. In plagioclase-hosted inclusions, variability in La/Yb decreases with decreasing host anorthite content. This is interpreted as evidence of concurrent mixing and crystallization of diverse primary melt compositions supplied to the Holuhraun magmatic system. As crystallization and mixing progress, compositional variability collapses to the mean melt inclusion and glass values, shown by the black and red lines, respectively. Note that the mean whole-rock La/Yb is identical to the mean melt inclusion La/Yb. Similar relationships between melt inclusion La/Yb and host mineral composition are observed in olivine-hosted melt inclusions from Laki (Neave et al. 2013; Hartley et al. 2014), Skuggafjöll (Neave et al. 2014) and Borgarhraun (Maclennan et al. 2003), and olivine- and plagioclase-hosted melt inclusions from older eruptions at Holuhraun (Hartley and Thordarson 2013). Compositions of plagioclase-hosted melt inclusions from the 10 ka Grímsvötn tephra series (Neave et al. 2015) are shown for comparison

Holuhraun matrix glasses have a mean La/Yb of 2.58 ± 0.77 (1*σ*) (Fig. 5). The high standard deviation is due to the heterogeneous glass compositions measured around large macrocrysts in sample H14. The H14 glasses range in La/Yb from 1.68 to 4.36, and La/Yb in these glasses is inversely correlated with MgO. Considering only the vesicular glass shards, the mean La/Yb is 2.34 ± 0.13.

The mean melt inclusion La/Yb is 2.44 ± 0.59 (1*σ*). Concentrations of incompatible trace elements such as La and Yb are negatively correlated with PEC-corrected melt inclusion MgO contents, as well as with host macrocryst compositions (Fig. S4). Most melt inclusion compositions cluster around the mean melt inclusion and glass La/Yb (Fig. 5; Fig. S5), although a small number of somewhat more enriched inclusions are hosted in An_>85_ plagioclases. The two most enriched inclusions (La/Yb > 4.7) are hosted in An_~78_ plagioclases, while the three most depleted inclusions (La/Yb < 1.8) are hosted in two Fo_86_ olivines and one An_75_ plagioclase (Fig. 5). Two inclusions, hosted in An_86_ and An_88_ plagioclases, are moderately depleted in high field strength elements with Zr/Y < 2.1 (Fig. 4a), and are similar in composition to HFSE-depleted inclusions from the 10 ka Grímsvötn tephra series (Neave et al. 2015). These inclusions have La/Yb = 3.44 and 3.88.

### Light lithophile elements

**Fig. 6:**
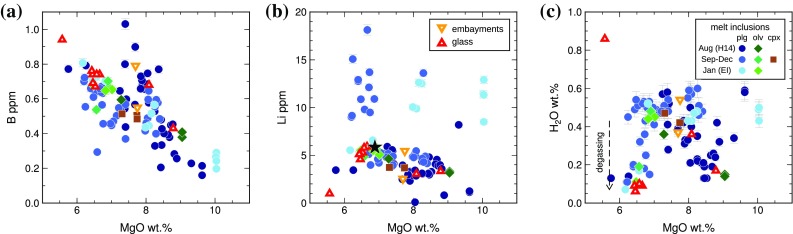
Light lithophile element and H_2_O concentrations of melt inclusions, embayments, and glasses from the 2014–2015 Holuhraun eruption. Melt inclusions have been corrected for post-entrapment crystallization. **a** Boron behaves as an incompatible trace element in the Holuhraun magma. A subset of inclusions, mostly from sample H14, have higher B contents at a given MgO content than the main population of inclusions. **b** Lithium appears to behave as an incompatible trace element in most melt inclusions. A sub-population of inclusions have high Li concentrations > 8 ppm. **c** H_2_O contents in Holuhraun melt inclusions show no systematic relationship with MgO, and cannot be explained by simple fractional crystallization. A small population of more evolved melt inclusions have either trapped a partially degassed melt or experienced post-entrapment dehydration. Vesicular tephra glasses have experienced syn-eruptive degassing and contain 0.07–0.08 wt% H_2_O

Boron contents in Holuhraun melt inclusions range from 0.16 to 1.03 ppm (mean 0.55 ± 0.17). Boron is negatively correlated with MgO, although a sub-population of inclusions, mainly from sample H14, have higher B contents at a given MgO than the main population (Fig. 6a). The matrix glasses range from 0.43 to 0.94 ppm B, with the highest and lowest B contents found in the glasses from sample H14. The vesicular tephra glasses contain 0.74 ± 0.04 ppm B (Fig. 6a).

The mean Li content of the Holuhraun matrix glasses is 4.03 ± 1.58 ppm, or 4.87 ± 0.94 ppm if only the vesicular tephra glasses are considered. The melt inclusions can be divided into two sub-populations with Li ≤ 8 and Li > 8 ppm (Fig. 6b). In the low-Li population, measured Li contents range from 1.13 to 6.57, and Li is weakly negatively correlated with MgO (*R*^2^ = 0.48). Melt inclusions in the high-Li population are hosted exclusively in plagioclase, and contain 8.18–18.12 ppm Li. These inclusions show no correlation between Li and MgO: Li is highly variable across the full compositional range (Fig. 6b).

## Discussion

### Fractional crystallization modelling

To test the ability of a single liquid line of descent (LLD) to reproduce the full suite of Holuhraun melt inclusion compositions, fractional crystallization models were calculated using Petrolog3 (Danyushevsky and Plechov 2011). Forward crystallization models were calculated for the mean composition of the three most primitive melt inclusions (10.0 wt% MgO) and for the mean composition of six inclusions with MgO from 8.72 to 9.05 wt%. We also tested reverse fractional crystallization models, taking the average vesicular tephra glass composition as representative of the Holuhraun carrier melt. Models were run to simulate crystallization at pressures of 0.001, 2, 4, and 6 kbar, which encompass likely pressures of crystallization within magmatic systems in Iceland’s Eastern Volcanic Zone (EVZ) (Neave and Putirka 2017), at an oxygen fucacity of FMQ + 0.2. Forward and reverse crystallization calculations were tested using a range of mineral–melt equilibrium models (Danyushevsky 2001; Langmuir et al. 1992; Gaetani and Watson 2002) for olivine, plagioclase, and clinopyroxene.

The calculated LLDs are sensitive to starting composition, pressure, and the choice of mineral–melt equilibrium models. In Fig. 3, we show LLDs calculated by forward crystallization of the primitive melt inclusion composition, using the mineral–melt equilibria of Langmuir et al. (1992) and with ${\text{Kd}}_{{{\text{Fe-Mg}}}}^{{{\text{ol-liq}}}}$ modelled after Toplis (2005). Overall, the Holuhraun melt inclusion data are not well reproduced by any single LLD. We computed several forward and reverse crystallization models varying the choice of starting composition, pressure, and mineral–melt equilibria, and in no case did the model LLD perfectly reproduce the pattern of the Holuhraun data. Given the variability in melt inclusion major element oxide contents at any given MgO, this is not a surprising result. Thus, while the compositional trends in Fig. 3 are broadly consistent with a dominant fractional crystallization control, we conclude that fractional crystallization alone is insufficient to explain the observed variability in melt inclusion compositions.

### Temporal evolution of melt inclusion compositions

There is no evidence of temporal evolution in the Holuhraun whole rock or tephra glass compositions (Fig. S6). The kernel density estimates in Fig. 3e indicate that the most probable MgO content for inclusions from sample H14 is higher than for inclusions collected later in the eruption (see also Fig. S7). However, the mean MgO content of the H14 melt inclusions is 8.13 ± 0.81 wt% (1*σ*), which is statistically indistinguishable from the 7.36 ± 0.99 wt% MgO for inclusions in samples collected between September and December 2014. Likewise, we find no statistically significant time-dependent variability in incompatible trace element ratios such as La/Yb in the Holuhraun melt inclusions (Fig. S9a). Over 40% of the inclusions analysed were from sample H14, so it is possible that any apparent temporal evolution in the melt inclusion compositions (or lack thereof) reflects a sampling bias that might not persist if more samples were analysed. We note that sample EI, collected on day 145 of the eruption, contains the most primitive inclusions at 10.0 wt% MgO as well some of the most evolved inclusions at 6.2–6.9 wt% MgO (Fig. 3e).

### Lithium in melt inclusions

Lithium appears to behave as an incompatible trace element in the main population of Holuhraun melt inclusions with Li < 8 ppm (Fig. 6b). However, no such relationship can be inferred for the high-Li (Li > 8 ppm) population. It is possible that the variability in melt inclusion Li content is time dependent: only one inclusion from sample H14 falls within the high-Li population, while the remainder are found in samples collected after the initial stages of the eruption (Figs. S8, S9b). It is very difficult to relate the high-Li population to the low-Li population by a simple fractional crystallization process. Thus, if the melt inclusions preserve the light lithophile element contents of their carrier melts at the time of inclusion trapping, this unusual compositional distribution could reflect either heterogeneous Li distribution in the Holuhraun melt, or post-entrapment modification of Li.

**Fig. 7:**
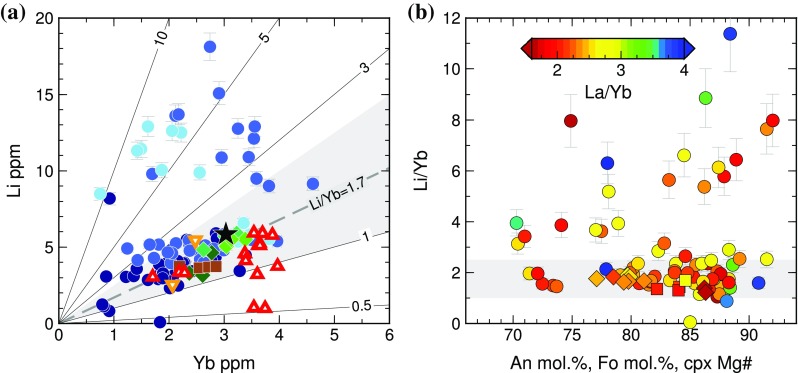
**a** Li vs. Yb for melt inclusions and glasses from Holuhraun, using the same symbols as Fig. 3. Lines show contours of constant Li/Yb. MORB is typified by Li/Yb ~ 1.7, and the grey-shaded area shows 1.0 < Li/Yb < 2.5 as obtained for olivine-hosted melt inclusions from Kilauea (Edmonds 2015). Most melt inclusions fall within this expected OIB range, with the exception of plagioclase-hosted inclusions with anomalously high Li > 8 ppm and some degassed matrix glasses. **b** Li/Yb vs. host macrocryst composition, coloured by La/Yb. The variability in Li/Yb decreases with decreasing host plagioclase anorthite content

Post-entrapment modification of melt inclusion Li contents can be assessed using the ratio Li/Yb. These trace elements are similarly incompatible in basaltic melts; thus, Li/Yb is expected to remain constant during fractional crystallization. Mid-ocean ridge basalt (MORB) glasses have Li/Yb values of ~ 1.7 (Jenner and O’Neill 2012). While typical ocean island basalt (OIB) glasses have mean Li/Yb values slightly greater than 1.7 (Ryan and Langmuir 1987), olivine-hosted melt inclusions from Kilauea, Hawaii, indicate that primary Li/Yb values in OIB range between 1.0 and 2.5 (Edmonds 2015). Seventy of the 77 Holuhraun melt inclusions with Li concentrations < 8 ppm have Li/Yb values within the expected range for OIB (Fig. 7a), with no systematic variability between Li/Yb and host macrocryst composition (Fig. 7b). Lithium in these inclusions therefore appears to be unaffected by post-entrapment modification. In contrast, all but two of the 22 high-Li inclusions have Li/Yb greater than the expected OIB values (Fig. 7a), suggesting that high-Li was not a primary feature of the trapped melt compositions. Furthermore, the maximum Li/Yb decreases with decreasing host plagioclase anorthite content (Fig. 7b), but increases continuously with eruption progress (Fig. S9).

For the high-Li inclusions, there is no statistically significant correlation between Li and any other incompatible or volatile element, including H_2_O (Figs. S6, S8). It is therefore unlikely that high Li concentrations resulted from the trapping of incompatible element-enriched boundary layers surrounding rapidly growing crystals (cf. Baker et al. 2008). High Li contents in melt inclusions could be achieved by diffusive exchange between an initially low-Li inclusion and a Li-enriched carrier melt: at magmatic temperatures, Li^+^ diffusion through plagioclase is approximately two orders of magnitude faster than H^+^ (Giletti and Shanahan 1997). However, the maximum measured Li in the Holuhraun matrix glass is 6 ppm, which is insufficient to produce high-Li melt inclusions by diffusive re-equilibration. It is possible that Li was heterogeneously distributed within the carrier melt, allowing melt inclusions either to trap Li-enriched melt pockets or to undergo diffusive exchange with melt containing up to 18 ppm Li. If this is the case, then the high-Li melt pockets would be expected to have degassed some 50% of their Li budget during ascent and eruption. While Li is generally considered to be a non-volatile element with a vapour–melt partition coefficient of less than 0.1 (Mather 2015, and references therein), investigations of trace metal degassing into the Holuhraun plume suggest that Li exhibited highly volatile behaviour during this eruption (Ilyinskaya et al. 2017). We therefore speculate that the high-Li melt inclusions were trapped during crystallization in Li-supersaturated melt pockets, which subsequently degassed any excess Li at the vents.

### Concurrent mixing and crystallization in the Holuhraun magmatic system

It has been shown that La/Yb variability in olivine-hosted melt inclusions decreases with decreasing host forsterite content in the products of several Icelandic eruptions (e.g., Maclennan et al. 2003; Maclennan 2008; Neave et al. 2013; Hartley et al. 2014). This is interpreted as evidence of concurrent mixing and crystallization of diverse mantle melt compositions delivered to these magmatic systems (Maclennan 2008).

While melt inclusions from Holuhraun range in La/Yb from 1.43 to 5.14, the most extreme enriched and depleted compositions are not found in the most primitive host crystals. For inclusions hosted in An_≥82_ macrocrysts, the minimum and maximum La/Yb are 1.63 and 3.88, respectively, and the 1*σ* standard deviation in La/Yb for these inclusions is 0.53. This is similar to the 1*σ* standard deviation in La/Yb for inclusions hosted in Fo_≥86_ olivines from Borgarhraun (0.57; Maclennan et al. 2003), but much lower than that of inclusions hosted in Fo_≥81_ olivines from Laki (0.98; Hartley et al. 2014). It is not straightforward to make direct comparisons between olivine- and plagioclase-hosted melt inclusion datasets, because the first appearance of these minerals on the liquidus is dependent on both primary melt composition and crystallization pressure. However, eruptions with higher mean La/Yb are expected to have a higher dispersion in melt inclusion La/Yb, even if the melt is better mixed (Maclennan 2008; Rudge et al. 2013). The fact that Holuhraun has a more enriched bulk composition than Borgarhraun, but has similar dispersion in melt inclusion La/Yb leads us to suggest that the diversity of primary melt compositions supplied to the Holuhraun magmatic system was more limited than for Borgarhraun. Alternatively, the Holuhraun melt inclusion record we have accessed may not capture the earliest stages of mixing between diverse mantle melts: it is possible that the earliest stages of mixing and crystallization were captured by melt inclusions in high-forsterite olivines, which are notably absent in Holuhraun erupted products.

It is useful to compare the Holuhraun plagioclase-hosted melt inclusion compositions with those from the 10 ka Grímsvötn tephra series (Neave et al. 2015), which is currently the only other extensive dataset of plagioclase-hosted melt inclusion compositions available for an Icelandic eruption. While Holuhraun and Grímsvötn melt inclusions have similar mean La/Yb of 2.4 and 2.1, respectively, the Grímsvötn melt inclusions are compositionally more diverse: the 1*σ* standard deviation in La/Yb is 0.98 for inclusions hosted in An_≥86_ plagioclases (relative to 0.53 for Holuhraun). The mean La/Yb for inclusions hosted in high-An plagioclases from the 10 ka Grímsvötn tephra series is also much lower than that of its carrier melt (La/Yb = 3.6). Neave et al. (2015) explained this observation by arguing that high-An macrocrysts from Grímsvötn crystallized from depleted, low-La/Yb melts, and were subsequently entrained in a more enriched, high-La/Yb melt during convective stirring in magma reservoirs. In contrast, the mean La/Yb of the Holuhraun melt inclusions is identical within error to its carrier melt; thus, a complex magmatic plumbing system involving the entrainment of depleted crystal mushes is not required to explain the first-order trace element variability observed in the Holuhraun melt inclusions.

### Pressures of melt inclusion crystallization

Having established that the Holuhraun melt inclusions preserve some record of concurrent mixing and crystallization of diverse primary melts, we next seek to establish where these processes occur within the magmatic system. Pressures of glass and melt inclusion equilibration can be estimated using the position of the olivine–plagioclase–augite–melt (OPAM) thermal minimum as a barometer (e.g., Grove et al. 1993), provided that the melt is saturated in all three phases. Yang et al. (1996) based their parameterisation of olivine–plagioclase–clinopyroxene–saturated experimental melt compositions on a multiple linear regression, where the cation fractions *X*_Mg_, *X*_Ca_ and *X*_Al_ were expressed as functions of pressure and other melt major element contents.

Crystallization pressures of multiply saturated natural samples can be estimated by visual inspection of projected compositions with respect to the position of the OPAM boundary predicted at a range of pressures (e.g., Yang et al. 1996; Maclennan et al. 2001). Michael and Cornell (1998) rearranged Eq. 2 of Yang et al. (1996) to calculate pressures of crystallization, but this rearrangement is not permitted for relationships derived from multiple linear regression. Kelley and Barton (2008) employed a more complex method: for each sample of interest, they calculated a series of liquid compositions along the olivine–plagioclase–clinopyroxene cotectic at 1 kbar intervals using the Yang et al. (1996) parameterisation, calculated the normative mineral components of these liquids, performed linear regression of pressure against the components for the calculated and original sample compositions, and then calculated a mean estimated equilibration pressure to be used for barometry. The extra steps of ternary projection and linear regression add unnecessary complication and uncertainty to the method. Furthermore, they were not always able to use petrographic and microanalytical measurements to verify that the assumption of multiple saturation in olivine, plagioclase and clinopyroxene was valid. This difficulty may have contributed to the large dispersion in pressure estimates that they found for several Icelandic volcanoes and eruptions. For example, Kelley and Barton (2008) obtained glass crystallization pressures of 1.4–7.7 kbar for Laki, utilising magmatic, phreatomagmatic and rootless cone tephras, glassy lava selvages, and glassy melt inclusion compositions from Thordarson et al. (1996). This is significantly greater pressure range than the 1–2 ± 1 kbar obtained by OPAM barometry using only magmatic tephra glasses from the same eruption (Neave et al. 2013).

Our approach is to use a statistical test to screen melt compositions for three-phase saturation, to have confidence that the OPAM equilibration pressures calculated according to the Yang et al. (1996) parameterization are robust. Measured melt compositions that closely match the *X*_*Mg*_, *X*_*Ca*_ and *X*_*Al*_ values predicted by equations 1–3 from Yang et al. (1996) calculated at a particular pressure are expected to fall on or near the OPAM boundary—in other words, the melt was saturated in olivine, plagioclase and clinopyroxene at that pressure. This is particularly useful for melt inclusion compositions where the crystallizing assemblage at the time of inclusion trapping can no longer be visually assessed. The first step is to calculate the χ^2^ misfit for *X*_*Mg*_, *X*_*Ca*_ and *X*_*Al*_ between the measured melt compositions and those predicted by the Yang et al. (1996) parameterisation. The Chi square is calculated using the standard expression:

$${\chi ^2}=\mathop \sum \limits_{{i=1}}^{3} {\left[ {\frac{{X_{i}^{o} - X_{i}^{Y}}}{{{\sigma _{{X_i}}}}}} \right]^2}$$

with $X_{i}^{o}$ the observed cation mole fractions of Mg, Ca, and Al, $X_{i}^{Y}$ those predicted from the Yang et al. (1996) parameterisation, and *σ* reflecting the analytical uncertainty on glass compositions measured by EPMA, which is taken to be ± 5% reflecting the expected analytical precision for major element oxide components (e.g., Neave et al. 2015). We iteratively solve equations 1–3 from Yang et al. (1996) at 0.01 kbar intervals between − 5 and 15 kbar, and the minimum *χ*^2^ value is found at the best-fitting model pressure. The second step is to estimate the quality of fit between the measured and predicted melt compositions using the *χ*^2^ vs. pressure distribution. This provides a significance estimate that we term the probability of fit *P*_F_ in the following discussion, which allows us to effectively screen the input melt compositions for three-phase saturation. A crucial point here is that many barometric estimates based on the Yang et al. (1996) parameterisation have not been carefully filtered to ensure that the melts are likely to have been in equilibrium with olivine, plagioclase and clinopyroxene. Using our approach, melts that are far from three-phase saturation, and therefore unsuitable for OPAM barometry, return a low probability of fit and can be excluded from further consideration. The petrography of the Holuhraun samples indicates that the melts were three-phase saturated over the compositional range of the inclusions presented here, with the possible exception of the most primitive inclusions.

#### Testing the method using experimental data

Our initial tests on the compositions of experimental charges generated by melting a natural sample of Reykjanes Ridge basalt (Yang et al. 1996) indicate that good *χ*^2^ fits can be found, returning probabilities of fit *P*_F_ > 0.9. However, the returned pressures are significant overestimates of the true experimental pressures, with a systematic overestimate of 1.4 kbar and a standard deviation of 0.4 kbar. Similarly, a multiply saturated low-H_2_O MORB composition equilibrated at an experimental pressure of 2.0 kbar (run 127 from Berndt et al. 2005) returned an OPAM pressure of 4.5 kbar and a poor probability of fit (*χ*^2^ = 31, *P*_F_ < 0.1). Experiments on Shatsky Rise samples conducted at pressures of 1–7 kbar (Husen et al. 2016) had generally poor *χ*^2^ fits, with only one of 41 experiments returning *P*_F_ > 0.9. For these data, the systematic pressure overestimate was 1.9 kbar, and for each defined experimental pressure there is significant variability in the returned OPAM pressure (typically *σ* = 2 kbar). However, the Shatsky Rise experiments tend to be on more alkaline and more hydrous compositions than either MORB or the Holuhraun melt inclusions and glasses. While the Shatsky Rise samples are classified as tholeiitic basalts (Husen et al. 2016), we note that the OPAM barometer should not be used for transitional or alkali basalt compositions.

Further tests were conducted using an expanded experimental dataset including all the experiments used to calibrate the Yang et al. (1996) parameterisation and a number of more recent studies (Toplis and Carroll 1995; Berndt et al. 2005; Thy et al. 2006; Feig et al. 2006, 2010; Botcharnikov et al. 2008; Whitaker et al. 2007, 2008; Husen et al. 2016), providing a total of 157 experiments on multiply saturated melt compositions (Fig. S10a). Our implementation of the Yang et al. (1996) parameterisation across this dataset yields a systematic pressure overestimate of 1.0 kbar, with a substantial random error of ± 2.5 kbar (Fig. S10b). When the error in the pressure estimate is plotted as a function of *P*_F_ according to the *χ*^2^ estimate, a clear pattern emerges. Around 60% of the experiments provide *P*_F_ < 0.8, where the ability of the Yang et al. (1996) parameterisation to simultaneously fit *X*_*Mg*_, *X*_Ca,_ and *X*_Al_ is poor (Fig. S10c). For the 40% of experiments with *P*_F_ > 0.8, we find a systematic pressure overestimate of 0.34 kbar and random error of ± 1.32 kbar (Fig. S10d). The results of these tests indicate that a reasonable strategy is to filter unknown data using the *χ*^2^ approach, and then to consider only the pressures that pass the exacting test *P*_F_ > 0.8. This approach enables us to identify and discard melt compositions that are not multiply saturated in olivine, plagioclase and clinopyroxene, or melt inclusion compositions that have been pulled away from the OPAM boundary by significant PEC around the host mineral walls. This minimises the systematic error in the returned pressure estimates. We note that some melt compositions that are three-phase saturated do not pass the *P*_F_ > 0.8 statistical filter, but these false negatives are not important for our purposes. The key objective in setting a high threshold at *P*_F_ > 0.8 is to eliminate false positives, where the melt composition is not three-phase saturated and the resultant OPAM pressure is erroneously high.

#### Application to Holuhraun melt compositions

**Fig. 8:**
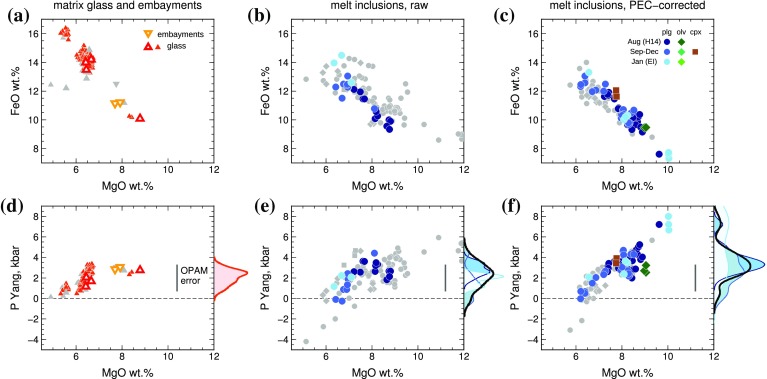
Application of the Yang et al. (1996) OPAM barometer to Holuhraun erupted products. The upper panels show the compositions of erupted products, and the lower panels show the equilibration pressures returned by OPAM barometry. **a, d** Holuhraun matrix glasses and embayments; **b, e** raw melt inclusion compositions; **c, f** melt inclusions compositions corrected for post-entrapment crystallization. In all plots, coloured symbols with show compositions where the returned probability of fit *P*_F_ is greater than 0.8, and grey symbols show compositions where *P*_F_ < 0.8. Kernel density estimates to the right of plots **d**–**f** show the relative probability of equilibration pressures for compositions with *P*_F_ > 0.8. The blue kernel density curves correspond to plagioclase-hosted inclusions collected on different dates; the black curve shows all melt inclusions with *P*_F_ > 0.8

We applied our implementation of the Yang et al. (1996) parameterisation to 186 analyses of Holuhraun glass: the ten samples analysed by SIMS in this study, and the compositions used to calculate the OPAM pressures reported by Gudmundsson et al. (2016). Acceptable fits with *P*_F_ > 0.8 were obtained for 134 analyses, returning a mean equilibration pressure of 2.1 ± 0.7 kbar (Fig. 8a, d). High-MgO glass compositions returned equilibration pressures of 3.0–3.5 kbar; two embayments with acceptable fits (*P*_F_ > 0.8) also returned equilibration pressures around 3.0 kbar.

Melt inclusion OPAM equilibration pressures were calculated using both raw and PEC-corrected compositions. Of the 105 available raw melt inclusion compositions measured by EPMA, 25 inclusions returned acceptable fits (*P*_F_ > 0.8); all of these inclusions were hosted in plagioclase (coloured symbols in Fig. 8b, e). The mean returned equilibration pressure for these 25 inclusions was 2.2 ± 1.1 kbar. There is some indication that the melt inclusions can be subdivided into two populations (Fig. 8e): one of more primitive inclusions (MgO > 7.5 wt%) with equilibration pressures of 3.1 ± 0.7 kbar and one of more evolved inclusions (MgO < 7.5 wt%) with equilibration pressures of 1.5 ± 0.9 kbar, although there is no clear boundary separating these populations.

Using PEC-corrected melt inclusion compositions, 63 of the 105 melt inclusions returned acceptable fits (*P*_F_ > 0.8; coloured symbols in Fig. 8c, f). The mean equilibration pressure is 3.5 ± 1.7 kbar, and there are two distinct peaks in the probability distribution of equilibration pressures, at 3.2 and 7.4 kbar (Fig. 8f). The maximum returned equilibration pressure increases systematically with the extent of PEC experienced by the melt inclusions, but the correlation between equilibration pressure and PEC is not statistically significant (*R*^2^ = 0.24; Fig. S13). An encouraging feature of the PEC-corrected equilibration pressures is that three olivine-hosted inclusions and three clinopyroxene-hosted inclusions also show acceptable fits, and return pressures that lie within the range obtained for PEC-corrected plagioclase-hosted inclusions. The consistency of pressure estimates from inclusions hosted in the three mineral phases involved in the parameterisation (Fig. S14) indicates that the assumption of multiple saturation is likely to be valid for these inclusions.

The highest equilibration pressures > 7 kbar are returned by six PEC-corrected melt inclusions with MgO > 9.5 wt% (Fig. 8f; Fig. S15). High-pressure melting experiments on Icelandic basalts suggest that olivine, plagioclase and clinopyroxene can coexist at 6–10 kbar (Fisk et al. 1980), and all six of these inclusions returned *P*_F_ > 0.98. However, we note that these inclusions have PEC corrections of 12–16% and their PEC-corrected compositions do not precisely match published compositions for Bárðarbunga glasses (Fig. 3). The apparent three-phase saturation of these inclusions may therefore be an artefact of the PEC correction, and the high equilibration pressures are treated with caution.

The remaining 57 inclusions have shallower trapping pressures with a peak in the probability distribution at 3.2 kbar. Kernel density plots subdivided by eruption date indicate that melt inclusion trapping pressures may have varied over time (Fig. 8f). Inclusions from 31 August 2014 (sample H14) have a peak in the probability distribution at 3.4 kbar with a subsidiary shoulder at 5.0 kbar; no inclusions from this sample return equilibration pressures shallower than 2.0 kbar. In contrast, inclusions in samples collected between September and November 2014 have peaks in the probability distribution at 0.7, 2.5, and 4.2 kbar. These differences are just resolvable within the ± 1.32 kbar uncertainty of the OPAM barometer. Finally, inclusions from 22 January 2015 (sample EI) return equilibration pressures around 3.0 and 7.5 kbar, but the probability distribution for this sample is not well defined due to the small number of samples (*n* = 8).

Equilibration pressures of 3.4–5.0 kbar for inclusions from sample H14 are consistent with trapping pressures of 4.1 kbar recorded by high-density CO_2_ fluid inclusions in macrocrysts from the same sample (Bali et al. 2018). Similarly, CO_2_-rich fluid inclusions in macrocrysts erupted from September 2014 onwards record trapping pressures of 2.5 kbar or less (Bali et al. 2018), consistent with the shallower melt equilibration pressures in these later samples. Although the difference in mean melt inclusion equilibration pressures between sample H14 and later samples is smaller than the estimated uncertainty on our implementation of the Yang et al. (1996) barometer, the distribution of equilibration pressures and fluid inclusion trapping pressures permits the suggestion that the first-erupted magmas entrained and transported plagioclase macrocrysts from deeper in the Holuhraun magmatic system than later-erupted products. Melt inclusions trapped at the very base of the Holuhraun magmatic system at > 7 kbar are sampled infrequently and have been recovered only in macrocrysts from the start and end of the eruption. However, the small number of inclusions (*n* = 6) means that it not possible to speculate whether this distribution is reliably indicative of changes in the magma supply over the course of the eruption.

A limitation of the Yang et al. (1996) parameterisation is that it does not take the oxidation state or Cr content of the melt into account. We therefore re-calculated the Holuhraun glass and melt inclusion equilibration pressures using a re-parameterisation of the OPAM barometer that takes into account melt Fe^3+^ and Cr contents, and is calibrated against experimental studies that were not included in our implementation (Voigt et al. 2017, and references therein). The pressures obtained from the Voigt et al. (2017) parameterisation agree with our implementation to within 0.1 kbar. This agreement is better than the calibration uncertainty associated with either barometer, which lends confidence to our returned OPAM equilibration pressures of 2.0–3.0 kbar for Holuhraun glasses and later-erupted melt inclusions. Furthermore, both OPAM models return equilibration pressures that are within error of the 2.5–3.5 kbar pressures returned by clinopyroxene–liquid barometry (Hartley et al. 2017).

Our returned OPAM pressures are shallower than the 3.6–5.0 kbar reported by Gudmundsson et al. (2016). These authors calculated OPAM equilibration pressures for Holuhraun glasses using the Kelley and Barton (2008) implementation (hereafter KB08). We tested KB08 using the same experimental data used to calibrate our implementation of the Yang et al. (1996) OPAM parameterisation. Pressures returned by the KB08 approach overestimated the true experimental pressures by an average 0.9 kbar (Figs. S11, S12). Taking this overestimate into account, the OPAM pressures reported by Gudmundsson et al. (2016) are reduced to 2.7–4.1 kbar. The shallowest pressures in this range are therefore within error of our calculated glass equilibration pressures. The remaining scatter in the glass pressures reported by Gudmundsson et al. (2016) may be due to the fact that 52 of the 184 Holuhraun glass compositions returned *P*_F_ < 0.8 indicating that they are unsuitable for OPAM barometry, yet these compositions are not screened out when the KB08 method is adopted.

Having established the pressures of melt mixing, crystallization, and melt inclusion trapping in the Holuhraun magmatic system, we next consider the timescales over which these processes occurred.

### H_2_O, Ce, and the diffusive re-equilibration of H_2_O: constraints on crystal residence timescales

**Fig. 9:**
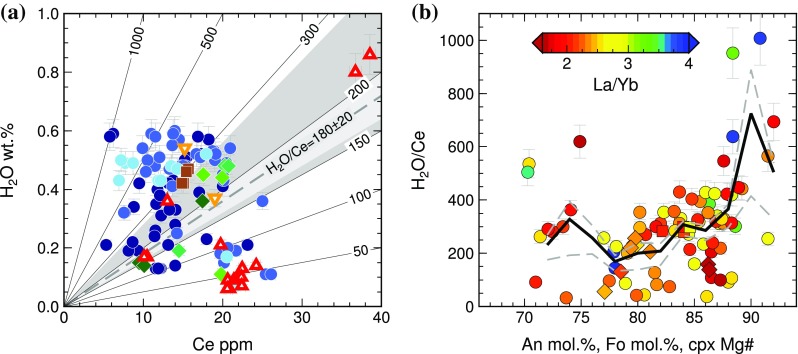
**a** H_2_O vs. Ce for melt inclusions and glasses from Holuhraun, using the same symbols as Fig. 3. Lines show contours of constant H_2_O/Ce. The light grey-shaded area shows the unmodified H_2_O/Ce = 180 ± 20 determined for Iceland’s Eastern Volcanic Zone (Hartley et al. 2015); the dark grey-shaded area indicates the expected range in H_2_O/Ce for undegassed MORB glasses, 150 < H_2_O/Ce < 280 (Michael et al. 1995). **b** H_2_O/Ce vs. host macrocryst composition, coloured by La/Yb. The black solid line shows the running average H_2_O/Ce for plagioclase-hosted melt inclusions (circles) calculated using a boxcar filter with bandwidth of 2 mol% An; dashed grey lines show the standard error of estimate for the filtered data

Recent studies of both natural and experimental samples have shown that at magmatic temperatures, the H_2_O contents of crystal-hosted melt inclusions may be modified by several weight percent on timescales of hours to days by diffusive re-equilibration of H^+^ between the melt inclusion and its external environment (e.g., Portnyagin et al. 2008; Gaetani et al. 2012; Bucholz et al. 2013; Hartley et al. 2015). Magmatic water contents at the time of melt inclusion trapping can be estimated using Ce as a proxy for H_2_O, if the undegassed H_2_O/Ce of the primary melt is known. Determining a primary H_2_O/Ce value for Holuhraun is challenging, since no melt inclusions or glasses in our study preserve H_2_O contents that can be unambiguously recognised as unmodified (Fig. 9a). The vesicular tephra glasses contain on average 0.1 wt% H_2_O and are considered to be degassed. However, the H_2_O contents of the non-vesicular glasses and embayments are positively correlated with K_2_O and Ce and negatively correlated with MgO, as expected for a relationship governed by fractional crystallization rather than degassing. If these samples are assumed to be representative of the undegassed carrier melt, then H_2_O/Ce for Holuhraun can be estimated at 253 ± 64 (1*σ*) (Fig. 9a). This is higher than the primary H_2_O/Ce value of 180 ± 20 previously estimated for the southern sector of the EVZ (Hartley et al. 2015), which may reflect regional heterogeneity in the Icelandic mantle source.

Melt inclusions with the highest H_2_O/Ce values are hosted in the most anorthitic plagioclases (Fig. 9b). The mean H_2_O/Ce decreases steadily for inclusions hosted in An_87_–An_78_ plagioclases, but rises slightly for inclusions hosted in An_77_–An_70_ plagioclases (Fig. 9b). We find no relationship between H_2_O/Ce and La/Yb. This contrasts with olivine-hosted melt inclusions from Laki and Skuggafjöll, where variation in H_2_O/Ce was controlled principally by primary melt variability: the most depleted inclusions experienced the greatest extent of diffusive hydration and had the highest H_2_O/Ce values (Hartley et al. 2015). In contrast, parental melts at Holuhraun are likely to have been more homogeneous, such that melt inclusion Ce contents at a given La/Yb are controlled by fractional crystallization rather than by mantle-derived variability.

The variable H_2_O contents and H_2_O/Ce values of Holuhraun melt inclusions suggest complex magmatic histories. High H_2_O/Ce in some inclusions (Fig. 9) may be taken as an indication of equilibration with a more hydrous carrier melt prior to eruption. In contrast, inclusions with low H_2_O contents and low H_2_O/Ce may either have trapped a partially degassed melt or experienced diffusive re-equilibration with a degassed carrier melt. It is possible that some of the Holuhraun melt inclusions preserve the magmatic H_2_O content at the time of inclusion trapping. However, by assuming that all the Holuhraun melt inclusions were trapped with initially primary H_2_O/Ce and that any modification of H_2_O/Ce was caused by post-entrapment H^+^ diffusion, it is possible to place minimum constraints on the residence timescales of host macrocrysts in different magmatic environments.

To estimate timescales of diffusive re-equilibration between melt inclusions and their external environment, we use a modified version of the model presented by Bucholz et al. (2013) to calculate an analytical solution for symmetric H^+^ diffusion through spherical crystals hosting spherical melt inclusions at their centres (Qin et al. 1992). Melt inclusion and host crystal dimensions were measured using a binocular microscope; the observed crystal shapes indicate that host crystals can be assumed to be whole crystals unaffected by syn-eruptive fragmentation. To model H^+^ diffusion in olivine, we used a partition coefficient ${\text{K}}_{{{{\text{H}}_{\text{2}}}{\text{O}}}}^{{{\text{olv-melt}}}}$ = 0.0007 (Le Voyer et al. 2014) and diffusion coefficient $D_{{{H^+}[001]}}^{{{\text{olv}}}}={10^{ - 1.4}}{\text{exp}}[ - (258/RT)]$ m^2^/s (Demouchy and Mackwell 2006). For plagioclase, we used a partition coefficient $K_{{{{\text{H}}_{\text{2}}}{\text{O}}}}^{{{\text{plg-melt}}}}$ = 0.01 (Hamada et al. 2011). The diffusivity of H^+^ in high-anorthite plagioclase is approximately one order of magnitude slower than for olivine (Johnson and Rossman 2013), but has not been quantified experimentally at temperatures > 1000 °C. We therefore estimated an appropriate diffusion coefficient $D_{{{{\text{H}}^+}}}^{{{\text{plg}}}}$ by extrapolating the model of Johnson and Rossman (2013) to higher temperatures.

At magmatic conditions of 3 ± 1 kbar and 1170 °C, PEC-corrected Holuhraun melt inclusions with (1) depleted compositions with La/Yb < 1.5 and initial H_2_O contents predicted assuming a primary H_2_O/Ce of 253 have water activities (*a*H_2_O) of ~ 0.020–0.053, and (2) enriched compositions with La/Yb > 3.5 have *a*H_2_O of ~ 0.025–0.145 (Burnham 1979). Taking the mean Ce content of the vesicular tephra glass (21.7 ± 1.3 ppm) and H_2_O/Ce = 253 ± 64, the undegassed Holuhraun carrier melt is predicted to contain 0.55 ± 0.17 wt% H_2_O, with a corresponding *a*H_2_O of $0.42_{{ - 0.19}}^{{+0.24}}$. Entrainment of melt inclusion-bearing macrocrysts into a carrier melt of this composition thus results in an *a*H_2_O gradient sufficient to facilitate diffusive over-hydration of the melt inclusions.

**Fig. 10:**
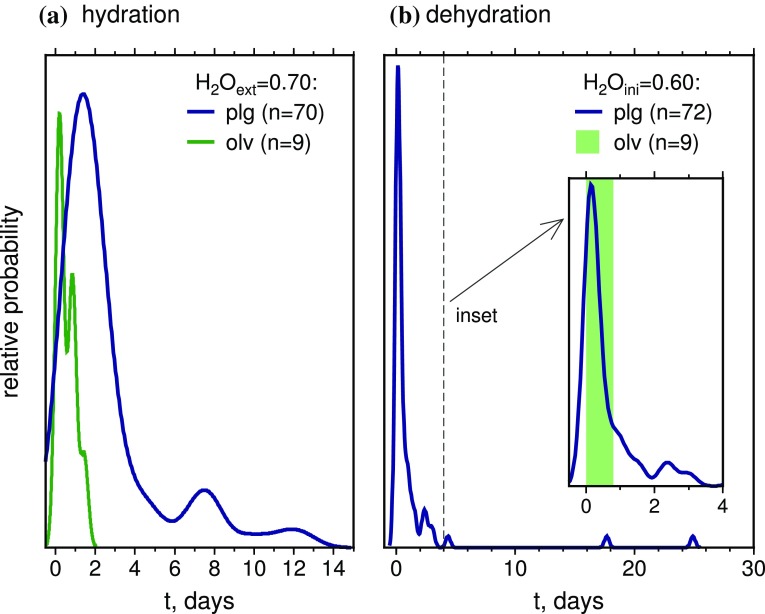
Kernel density estimates of calculated diffusive re-equilibration timescales for plagioclase-hosted melt inclusions. **a** Diffusive over-hydration of melt inclusions. Calculations assume that melt inclusions with initial primary H_2_O/Ce = 253 equilibrated with a melt composition identical to the carrier melt at 1170 °C. Timescales were modelled assuming an H_2_O content of 0.7 wt% for the external carrier melt, predicted using the measured Ce content of the most enriched melt inclusion. **b** Diffusive dehydration of melt inclusions. Calculations assume that melt inclusions initially in equilibrium with an undegassed carrier melt partially re-equilibrated with a degassed carrier liquid containing 0.08 wt% H_2_O at 1150 °C. On each plot, the vertical bars show the range of diffusion timescales obtained for olivine-hosted melt inclusions calculated under the same conditions

Eight melt inclusions have measured H_2_O > 0.55 wt%, with the highest measured H_2_O content being 0.6 wt%. In addition, 27 melt inclusions have measured H_2_O/Ce > 317, i.e., higher H_2_O contents than can be explained by primary melt heterogeneity alone. These values suggest that some inclusions were stored for part of their history in a melt that was more hydrous than the Holuhraun carrier melt, and gained H_2_O by diffusive hydration. The most evolved melt inclusion, with Ce = 27.6 ppm, can be used to estimate a maximum H_2_O content of 0.70 ± 0.17 wt% for this evolved carrier melt. We therefore calculated diffusive over-hydration timescales for each melt inclusion, assuming an external melt H_2_O content of 0.70 wt% and using the measured Ce content to calculate the initial H_2_O content, where primary H_2_O/Ce = 253. Calculations were performed at 3 kbar and 1170 °C, and were terminated when the melt inclusions reached equilibrium with the external melt. The calculated re-equilibration timescales for olivine- and plagioclase-hosted inclusions in a carrier melt containing 0.70 wt% H_2_O ranged from 0.4 to 12.5 days, with a peak in the probability distribution at 1.4 days (Fig. 10a). These calculations assume that all the melt inclusions were stored in, and completely re-equilibrated with, a hydrous external melt at some point in their history. While it is not possible to constrain whether or not individual inclusions experienced these magmatic conditions prior to eruption, the calculated timescales provide some indication of minimum crystal residence durations within the Holuhraun magmatic system.

The two longest melt inclusion hydration timescales of 11–12 days are comparable with the 14 days of pre-eruptive seismicity on the Bárðarbunga volcanic system (Sigmundsson et al. 2015). The melt transport rate is likely to be faster than this once an open dyke pathway exists between Bárðarbunga and the eruption site, and this may be reflected in the more typical minimum crystal residence timescales of 1–3 days. However, there is no difference in the distribution of minimum crystal residence timescales between sample H14 from 31 August 2014 and samples collected later in the eruption. This suggests that some crystals may have been entrained into the Holuhraun melt only a short time before eruption, and highlights a degree of chemical disequilibrium between the carrier melt and the macrocrysts it carried to the surface. Erupted products from the Bárðarbunga volcanic system commonly carry large plagioclase macrocrysts that exhibit chemical and isotopic disequilibrium with their carrier melt (Hansen and Grönvold 2000; Halldórsson et al. 2008). While it is expected that some melt inclusion-bearing plagioclases did crystallize directly from parental melts of the Holuhraun magma, it is not possible to definitively identify these macrocrysts from our current data.

For melt inclusions with H_2_O/Ce ≪ 253, we calculated timescales of diffusive H^+^ loss assuming that these inclusions were transported in a degassed carrier melt containing 0.08 wt% H_2_O (the measured H_2_O content of the most degassed vesicular tephra glasses) during ascent and eruption. Diffusive dehydration most probably occurred during subsurface magma transport, since post-eruptive re-equilibration of melt inclusions in rapidly quenched tephra samples can be assumed to be negligible. Dehydration modelling was conducted assuming that the inclusions were initially in equilibrium with a final carrier melt containing 0.60 wt% H_2_O. This melt H_2_O content is selected because (a) it is within the range of predicted carrier melt H_2_O contents for Holuhraun, and (b) it is the measured H_2_O content of the most hydrous melt inclusion. Dehydration calculations were performed at atmospheric pressure and 1150 °C, and were terminated when melt inclusions reached their measured H_2_O contents. Petrologically, this means that the melt inclusions ascended to the surface fast enough to prevent complete diffusive dehydration, and permits some inclusions to retain high H_2_O contents and high H_2_O/Ce acquired during a past episode of diffusive hydration.

Olivine-hosted inclusions with measured H_2_O/Ce < 253 would have required 0.02–0.8 days to reach their measured H_2_O contents by partial re-equilibration with a degassed carrier melt (Fig. 10b). Dehydrated plagioclase-hosted inclusions would have taken between 0.02 and 25 days to reach their measured H_2_O contents, with the peak in the probability distribution being 0.14 days (Fig. 10b). The two inclusions with calculated dehydration timescales > 5 days have measured H_2_O contents of 0.07 and 0.08 wt%. Both inclusions are from sample EI, which consists of scoria clasts collected from the side of the main eruptive vent. It is possible that these inclusions were thermally insulated and underwent diffusive dehydration over several days. However, other inclusions from the same sample preserve H_2_O contents up to 0.50 wt% and H_2_O/Ce > 253. We therefore suggest that the low-H_2_O inclusions formed at a late stage of crystallization and trapped an already degassed melt, which is consistent with these inclusions being located close to the rims of their host macrocrysts.

Given the uncertainty regarding the H_2_O content of the carrier melt(s) in which macrocrysts and microphenocrysts resided within the Holuhraun magmatic system, and the rapidity of H^+^ diffusion in both olivine and plagioclase, diffusive re-equilibration of H^+^ in melt inclusions provides only a minimum estimate of crystal residence timescales in the Holuhraun carrier melt. At present, it is not possible to constrain maximum crystal residence timescales in the Holuhraun magma, nor durations of crystal storage in magma chambers or crystal mush horizons prior to entrainment. Future studies will therefore focus on major and trace element diffusion chronometry in olivine and plagioclase to establish the provenance and magmatic histories of these crystals.

It is possible to use our calculated dehydration timescales to estimate the rates at which crystals moved to the surface after their carrier melt crossed the pressure of water saturation. Closed-system degassing has been argued to be significant in basaltic magmatic systems (e.g., Allard et al. 2005; Lloyd et al. 2014; Ferguson et al. 2016). Volatile saturation models (Newman and Lowenstern 2002; Witham et al. 2012) predict that the Holuhraun carrier liquid becomes water-saturated at pressures between ~ 1.0 and 0.4 kbar during closed-system degassing, with the variability arising from the choice of initial magmatic CO_2_ content and hence the mass percent of pre-existing exsolved vapour present. Assuming an initial volatile budget of 3000 ppm CO_2_ and 0.6 wt% H_2_O, the Holuhraun melt will begin to degas H_2_O at 1.0 kbar, but significant H_2_O loss only occurs at pressures below ~ 0.4 kbar. Combining these pressure estimates with diffusive dehydration timescales of 0.02–25 days and an average crustal density of 2.86 × 10^3^ kg m^−3^ allows us to calculate indicative magma ascent rates for the upper part of the conduit of between 0.001 and 1.7 m s^−1^, with the most probable dehydration timescales corresponding to ascent rates of 0.12–0.29 m s^−1^. These estimates represent minimum ascent rates, since we do not take into account the likelihood of H_2_O supersaturation and disequilibrium degassing during ascent. Our estimates are significantly slower than the magma transfer rate of 0.75 m s^−1^ obtained from radioactive disequilibria in Holuhraun gas phase samples (Gauthier et al. 2016). However, the rate of 0.75 m s^−1^ is based on the outgassing of volatile radionuclides upon early CO_2_ loss at depth within the magmatic system, and thus represents a different stage of magma ascent than has been considered in our calculation. Our calculated ascent rates are slower than the 0.05–0.45 MPa s^−1^ (~ 1.7–15 m s^−1^) obtained for various eruptions at Kilauea, Hawaii (Ferguson et al. 2016), but our higher estimates are comparable with ascent rates of ~ 0.2–2.0 m s^−1^ modelled for Stromboli (e.g., Pichavant et al. 2013).

### Comparison of petrological and geophysical datasets

**Fig. 11:**
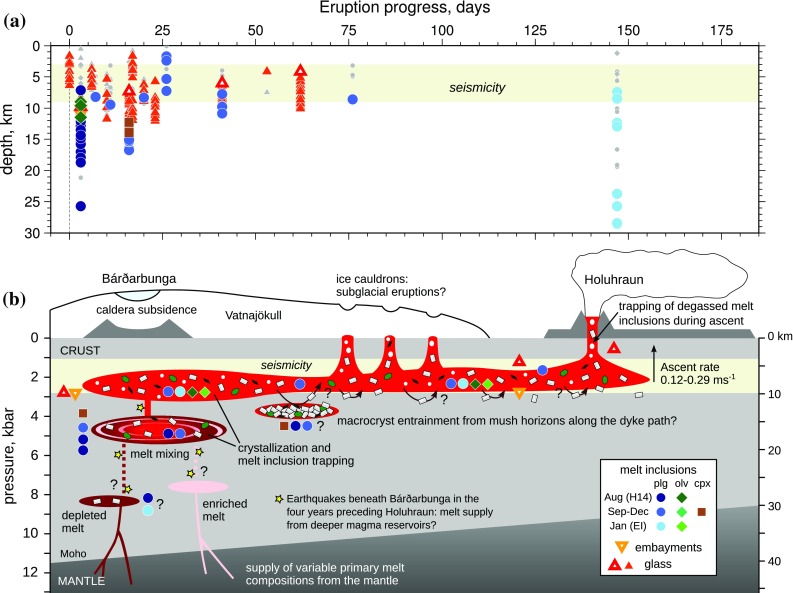
**a** Comparison of melt inclusion and glass OPAM equilibration depths with the depth of laterally propagating seismicity between Bárðarbunga and the eruption site. The coloured symbols show melt compositions where the returned probability of fit *P*_F_ to the Yang et al. (1996) OPAM barometer is greater than 0.8, and grey symbols show compositions where *P*_F_ < 0.8. OPAM equilibration pressures were converted to depths assuming a crustal density of 2.86 × 10^3^ kg m^−3^. **b** Schematic illustration showing how the Holuhraun magma may have been assembled and transported. Caldera subsidence at Bárðarbunga is likely related to magma draining from a shallow (2–3 kbar) magma reservoir into a lateral dyke. Three ice cauldrons along the dyke path most probably indicate the sites of small subglacial eruptions (Reynolds et al. 2017). Coloured symbols indicate the possible origins of melt inclusion- and embayment-bearing macrocrysts at the relevant pressures of crystallization. Macrocrysts may have been transported from magma reservoirs beneath Bárðarbunga, or entrained from mush horizons along the dyke path

Melt inclusion and carrier melt equilibration and trapping depths are compared with the depths of pre-eruptive seismicity in Fig. 11a. Equilibration depths for the carrier melt are comparable with the 3–9 km depth range of the pre-eruptive seismicity (Sigmundsson et al. 2015). The most probable melt equilibration pressure, 2.1 ± 0.7 kbar, corresponds to a depth of 7.5 ± 2.5 km and is in good agreement with the most common earthquake hypocentral depth of 6 ± 1 km (Ágústsdóttir et al. 2016). Taken at face value, melt barometry is thus consistent with models put forward involving lateral magma transport from Bárðarbunga volcano to the eruption site in a shallow- to mid-crustal dyke (Gudmundsson et al. 2016).

Some PEC-corrected melt inclusion equilibration depths cluster around 10 km (Fig. 8f), towards the base of the inferred lateral intrusion, but many equilibrate somewhat deeper (Fig. 11a). There appears to be a progressive shallowing of melt inclusion equilibration pressures over the course of the eruption (Figs. 8, 11a), which could be indicative of differences in the efficiency of crystal entrainment during the initial stages of melt ascent. Dyke propagation may have mobilised crystals from mush horizons beneath Bárðarbunga and/or from near the base of the magma intrusion at any point along the dyke path. As the eruption progressed and an open magma pathway was established, crystal entrainment may have been inhibited by viscous drag at the base of the dyke.

Melt inclusions from samples collected during the first 20 days of the eruption have equilibration depths ranging from 7.1 to 25.7 km, with most inclusions equilibrating significantly deeper than all but the very deepest earthquakes associated with dyke propagation (Fig. 11a). Regional geodetic modelling of caldera subsidence at Bárðarbunga implies a deflating source at 8–12 km (Gudmundsson et al. 2016) which provides an independent estimate of the likely depth of magma storage beneath Bárðarbunga. While the most probable melt inclusion equilibration depth of 11.4 km falls towards the lower end of this range, exactly half of the melt inclusions return equilibration depths in excess of 12 km. This implies that their host macrocrysts crystallized and trapped their inclusions at deeper levels in the crust, and were transported into the Bárðarbunga magma reservoir prior to the onset of seismicity on 16 August 2014. The occurrence of deep (12–25 km) earthquakes in the area east of Bárðarbunga over the 4 years preceding the Holuhraun eruption (Vogfjörð et al. 2015; Hudson et al. 2017) could be consistent with magma supply from depth to a reservoir 8–12 km beneath Bárðarbunga (Fig. 11b). Intriguingly, our maximum equilibration depths of 23.7–28.6 km match very closely the greatest depths of seismicity, which lends some confidence to these results.

Using geochemical and petrological data to reconstruct the magmatic systems that feed fissure eruptions is subject to a number of limitations. For example, while melt barometers such as that of Yang et al. (1996) are able to resolve depths of melt storage and crystallization within the uncertainty of the chosen barometer, they cannot resolve melt transport pathways in the lateral (i.e., horizontal) dimension. In the absence of geophysical and geodetic constraints on the nature of melt supply and transport, the simplest interpretation of our petrological and geochemical data would be that the Holuhraun eruption was fed via a vertically stacked series of interconnected magma reservoirs. Furthermore, while our data are consistent with the Holuhraun carrier melt being sourced beneath Bárðarbunga, we cannot definitively identify the position along the magma transport pathway at which melt inclusion-bearing macrocrysts were entrained. Our calculated diffusive hydration timescales for Holuhraun melt inclusions provide robust minimum residence timescales for host macrocrysts in the Holuhraun carrier melt on the order of 1–12 days. Diffusion chronometry in zoned melt inclusion-bearing macrocrysts may provide further constraints on whether macrocryst residence timescales in the carrier melt coincide with the onset of seismicity and lateral melt transport through the volcanic system, or whether these macrocrysts were entrained close to the eruptive vents with correspondingly short residence timescales.

## Conclusions

The 2014–2015 Holuhraun eruption was one of the best-monitored basaltic fissure eruptions ever to have occurred. We have used major, light lithophile, trace and rare-earth element data from crystal-hosted melt inclusions, embayments, and matrix glasses to investigate melt mixing, crystallization timescales, and transport processes in the Holuhraun magmatic system. Holuhraun melt inclusion and glass compositions are similar to Holocene erupted products from the Bárðarbunga volcanic system, and near-identical to the products of two older eruptions at Holuhraun. The range in major element compositions of Holuhraun melt inclusions, corrected for post-entrapment crystallization, resembles a liquid line of descent, although fractional crystallization alone is unable to explain the observed compositional diversity. Olivine–plagioclase–augite–melt barometry returns melt inclusion equilibration pressures between 0.5 and 8.0 kbar. A significant peak in the probability distribution at 3.2 kbar indicates the most likely pressure of melt storage, crystallization, and inclusion trapping. The mean matrix glass equilibration pressure of 2.1 ± 0.7 kbar is in close agreement with pressure estimates derived from clinopyroxene–liquid barometry, and within error of the depths of earthquakes between Bárðarbunga central volcano and the Holuhraun eruption site.

The diversity in incompatible trace element ratios such as La/Yb in Holuhraun melt inclusions is best explained by the concurrent mixing and crystallization of diverse primary melts. The absolute variability in melt inclusion compositions appears to be more restricted than has been inferred for other Icelandic eruptions, although it is possible that the Holuhraun melt inclusion record does not capture the earliest stages of melt mixing and crystallization. Diffusion chronometry based on H^+^ re-equilibration between melt inclusions and a more hydrous carrier melt indicates minimum residence times of 1–12 days for melt inclusion-bearing macrocrysts in the Holuhraun carrier melt. Similarly, diffusive dehydration of H_2_O-rich melt inclusions stored in a less hydrous carrier melt occurred over typical timescales of 0.14–0.16 days. This corresponds to indicative magma ascent rates of 0.12–0.29 m s^−1^, assuming closed-system degassing with H_2_O saturation occurring around 1.0–0.4 kbar beneath the vents.

Our petrological and geochemical data are consistent with lateral magma transport from Bárðarbunga to the eruption site in a shallow- to mid-crustal dyke, as has been interpreted using seismic and geodetic data. However, the simplest petrological explanation for the observed geochemical data is one of subvertical magma transport, since our petrological data cannot yet resolve magma transport in the lateral dimension. By combining robust melt inclusion equilibration pressures with diffusion chronometry in zoned magmatic crystals, it may be possible to deduce whether macrocrysts in eruptions of this type were transported laterally through the volcanic system over significant distances within a narrow pressure interval, or were instead entrained from crystal mush horizons close to the vents. Crucially, our results mark a significant step forward in reconciling interpretations of petrological and geophysical datasets for major volcano-tectonic episodes, and provides a vital framework for the interpretation of premonitory seismic and geodetic data in volcanically active regions.

### Electronic supplementary material

Below is the link to the electronic supplementary material.

Supplementary material 1 (PDF 16068 KB)

Supplementary material 2 (XLS 166 KB)

## Appendix

An example Python script using our implementation of the OPAM barometer is available from the authors on request.
