# Supplementary material for: Melt inclusion constraints on petrogenesis of the 2014–2015 Holuhraun eruption, Iceland
Source: Contrib Mineral Petrol. 2018 Jan 12;173(2):10. doi: 10.1007/s00410-017-1435-0 (PMC6953965; doi:10.1007/s00410-017-1435-0)
Supplement: Supplementary file 1 — Supplementary material 1 (PDF 16068 KB) [file 410_2017_1435_MOESM1_ESM.pdf]

Melt inclusion constraints on petrogenesis of the 2014-2015 Holuhraun eruption, Iceland

Supplementary information and figures

ME Hartley, E Bali, J Maclennan, DA Neave, SA Halldórsson

1. Analytical methods

1.1 Secondary ion mass spectrometry (SIMS)

Volatile, trace and rare earth elements were measured in melt inclusions and matrix glasses using the Cameca ims-4f ion microprobe at the University of Edinburgh, over three analytical sessions between March and July 2015. Carbon was measured separately from other volatile and trace elements, and samples were only carbon-coated for electron microprobe analysis once the SIMS measurements were completed.

Crystal-hosted melt inclusions were mounted in epo-thin epoxy resin, polished with diamond pastes, and gold coated. Samples were placed under high vacuum and allowed to outgas for 24 hours prior to analysis. Carbon analyses were performed first, using the same analytical procedure as described by Hartley et al. (2014).

Trace elements, H_2O and F were measured using a 15 kV primary O- beam with accelerating voltage of 4500 V minus an offset of -75±5 V, a 5±1 nA beam current and a 20±5 μm image field. Measurements were made with a spot size of ~20 μm centred in the pit made during the preceding C analyses.

The following isotopes were analysed in each cycle of a 10-cycle run, with counting time in seconds given in parentheses: ^{1}H (4), ^{7}Li (4), ^{11}B (4), ^{19}F (4), ^{30}Si (2), ^{35}Cl (5), ^{39}K (2), ^{47}Ti (2), ^{88}Sr (3), ^{89}Y (3), ^{90}Zr (3), ^{93}Nb (5), ^{138}Ba (3), ^{139}La (3), ^{140}Ce (3), ^{141}Pr (3), ^{143}Nd (3), ^{149}Sm (5), ^{151}Eu (8), ^{154}Gd (2), ^{156}Gd (2), ^{157}Gd (5), ^{159}Tb (5), ^{161}Dy (5), ^{165}Ho (8), ^{167}Er (8), ^{169}Tm (8), ^{171}Yb (8), ^{175}Lu (8). Peak positions were verified before each analysis. Masses 0.7 and 130.5 were measured as background for 1 and 5 seconds respectively in each cycle and were always zero. Counts were normalised to ^{30}Si, and absolute elemental concentrations were calculated by normalising ^{30}Si to the SiO_2 content of each melt inclusion or glass, determined subsequently by EPMA.

Oxide interference was monitored by measurement of ^{154}BaO/Ba and ^{156}CeO/Ce. Neither ratio varied systematically with time. The value of ^{156}CeO/Ce was used to estimate an effective offset voltage for each analysis which was then used in the calculation of interference corrections. The average offset voltage was 77±3 V.

Ion yields were assessed by repeat analyses of NIST-SRM610. For each session, ion yields from the first morning of analytical work were used to calibrate the melt inclusion data.

Repeat analyses were performed each morning and on at least one further occasion each day. These measurements were used to monitor analytical drift, which was shown to be insignificant for each session.

For each element, measured counts were used construct calibration curves for each trace element (for example, $^{139}La/^{30}Si$ vs. La) using a suite of basaltic glass standards with known trace element abundances. The calibration curves were then used to calculate trace element concentrations in the melt inclusion and glass samples from their measured counts per second.

Precision and accuracy were monitored by repeat analyses of natural and synthetic glass standards BCR-2G, GSA-1G, GSD-1G and KL2-G. Precision estimates were ±30% for Cl; ±20% for F; better than ±10% for the low abundance heavy rare earth elements (Yb, Lu); and better than ±2% for trace elements in high abundance (e.g. Zr, Y, La, Li). Estimated accuracy was 30% for Cl; 20% for B; 14% for F; between 2% and 6% for Nb, Pr, Eu, Tm and Lu; and better than 2% for all other elements.

For H_2O, the first 5 cycles of each analysis were discarded to minimise the effects of surface contamination. Background counts were monitored by analysing anhydrous standards GSD-1G, BCR-2G and matrix glasses known to be almost completely degassed. The average background correction was equivalent to 0.05 wt.% H_2O.

1.2 Electron probe microanalysis (EPMA)

Subsequent to the SIMS measurements, electron microprobe analyses were performed using the Jeol JXA-8230 Superprobe at the Institute of Earth Sciences, University of Iceland, using an operating potential of 15 keV, beam current of 10 nA, and spot size of 10 μm. Occasionally the spot size was reduced to 5 μm in order to analyse very small melt inclusions. Major and minor elements were measured in melt inclusions, matrix glasses and host macrocryst phases; volatiles S and Cl were also measured in the melt inclusions and glasses. Element concentrations were determined in WDS mode with peak counting time between 20 and 100 seconds depending on the element abundance and instrument sensitivity. Background count times were equal to the peak counting times; for elements such as S and Cl both higher and lower backgrounds were measured. Internal data reduction was performed using the CITZAF quantitative correction program (Armstrong, 1991).

A small number of matrix glasses were measured by Cameca SX100 electron microprobe at the University of Cambridge, using an operating potential of 15 keV, beam current of 10 nA and spot size of 10 μm. Major, minor and volatile (S, F, Cl) element concentrations were determined in WDS mode with peak counting time between 20 and 60 seconds depending on element abundance. The background count time was half the peak count time, or equal to the peak count time for minor elements such as phosphorous. Internal data reduction was performed using the inbuilt Cameca X-Phi PeakSight software.

For both electron probe instruments, estimated 1σ precision based on repeat measurement of standards was better than ±1% for SiO_2, TiO_2 and K_2O; better than ±2% for Al_2O_3,

FeO, MgO and CaO; and better than ±5% for all other major and minor elements.

2. Post-entrapment crystallization correction

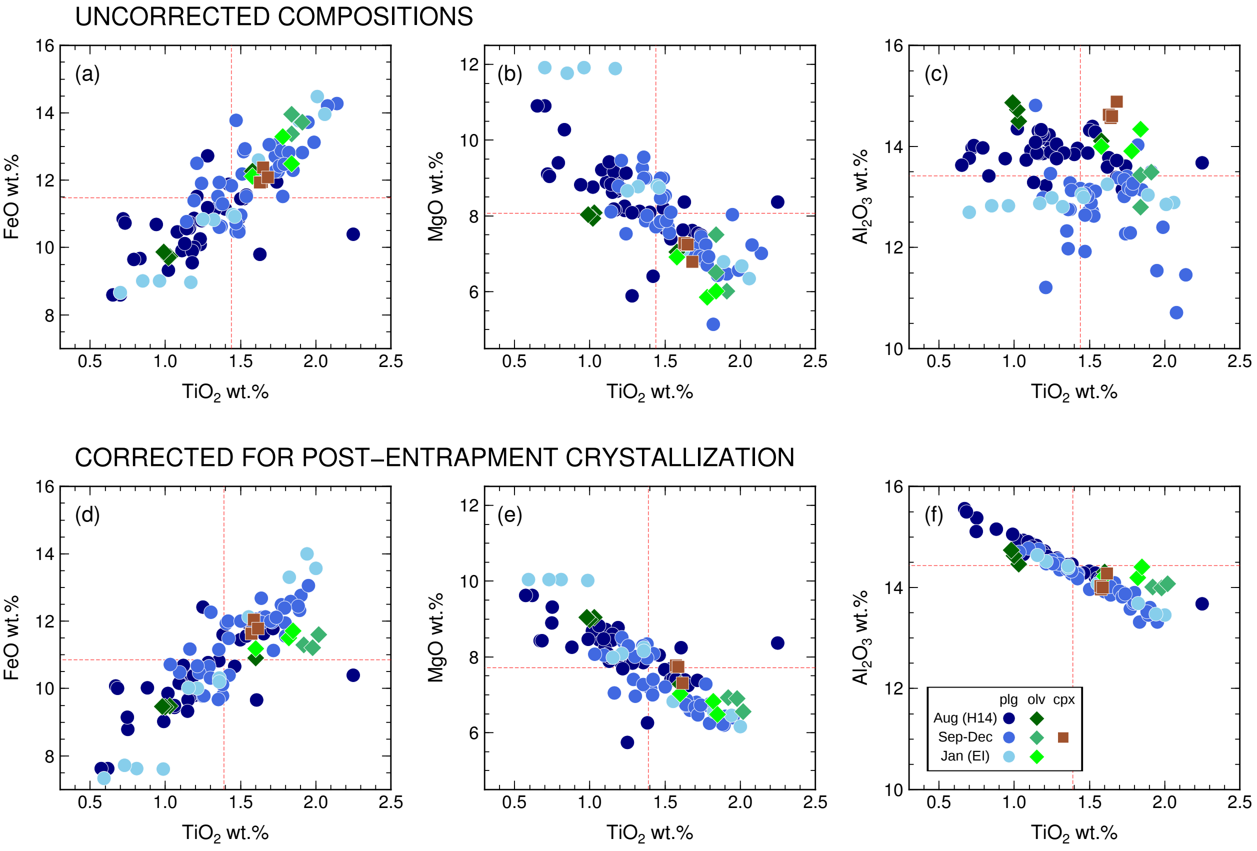

Figure S1: Comparison of (a-c) measured and (d-f) post entrapment crystallization-corrected melt inclusion compositions. Red dashed lines in each plot show the average melt inclusion composition. Olivine-hosted melt inclusion compositions were corrected for PEC using Petrolog3 (Danyushevsky and Plechov, 2011). For plagioclase-hosted inclusions, the composition of plagioclase measured adjacent to the inclusion was incrementally added back into the inclusion until the Al_2O_3 content at a given TiO_2 matched the Al_2O_3 vs. TiO_2 array defined by the olivine- and clinopyroxene-hosted melt inclusions.

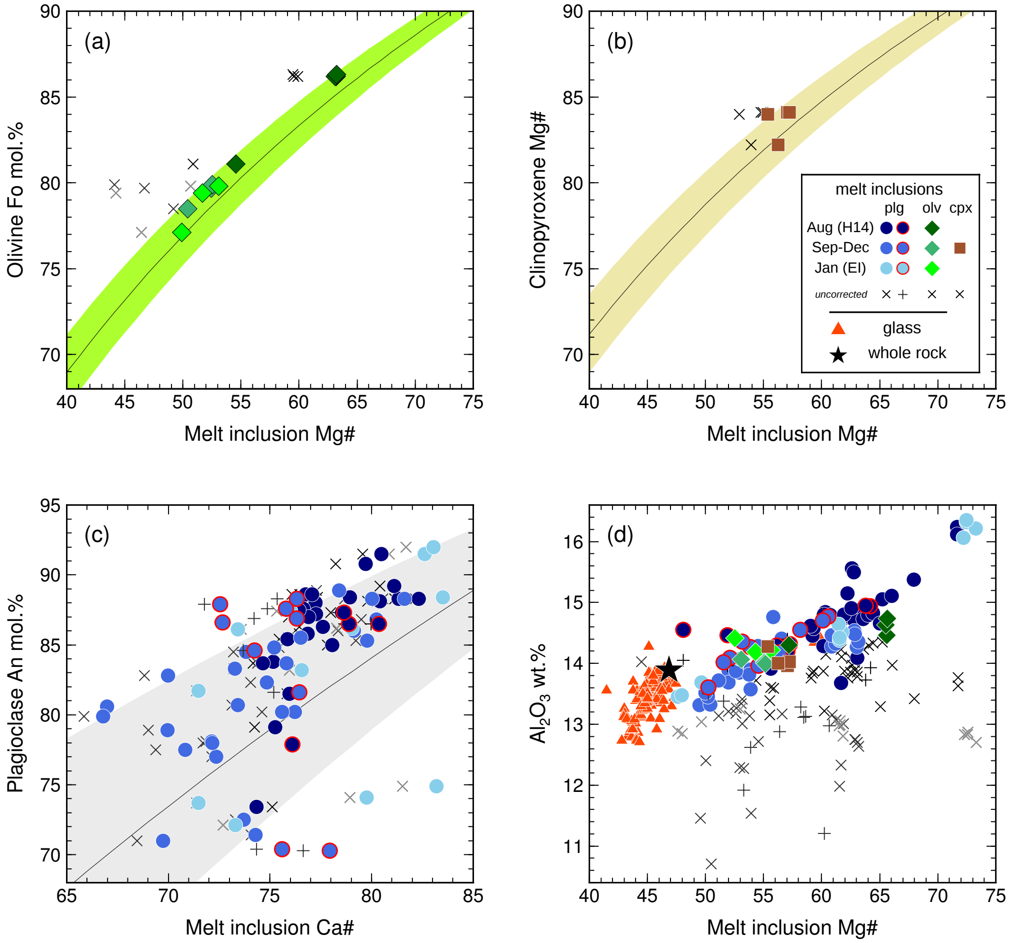

Figure S2: Measured and PEC-corrected melt inclusion compositions with respect to the host mineral composition. Filled symbols show PEC-corrected compositions. Symbols with red outlines indicate inclusions regarded as secondary or pseudo-secondary; all other inclusions are regarded as primary inclusions according to the definitions of Roedder (1984). (a) PEC-corrected compositions of olivine-hosted melt inclusions are in equilibrium with their host olivines. Green shading shows olivine-liquid pairs where Kd^{ol-liq}_{Fe-Mg}=0.30±0.03 (Roeder and Emslie, 1970). (b) PEC-corrected compositions of clinopyroxene-hosted melt inclusions are in equilibrium with their host clinopyroxenes. Light brown shading shows clinopyroxene-liquid pairs where $Kd^{cpx-liq}_{Fe-Mg}$=0.27±0.03 (Putirka, 1999). (c) PEC-corrected compositions of plagioclase-hosted melt inclusions. Grey shading shows where $Kd^{plg-liq}_{An-Ab}$=0.27±0.11 (Putirka, 2008). (d) Comparison of measured and PEC-corrected melt inclusion compositions. Holuhraun glass and whole-rock compositions are shown for reference.

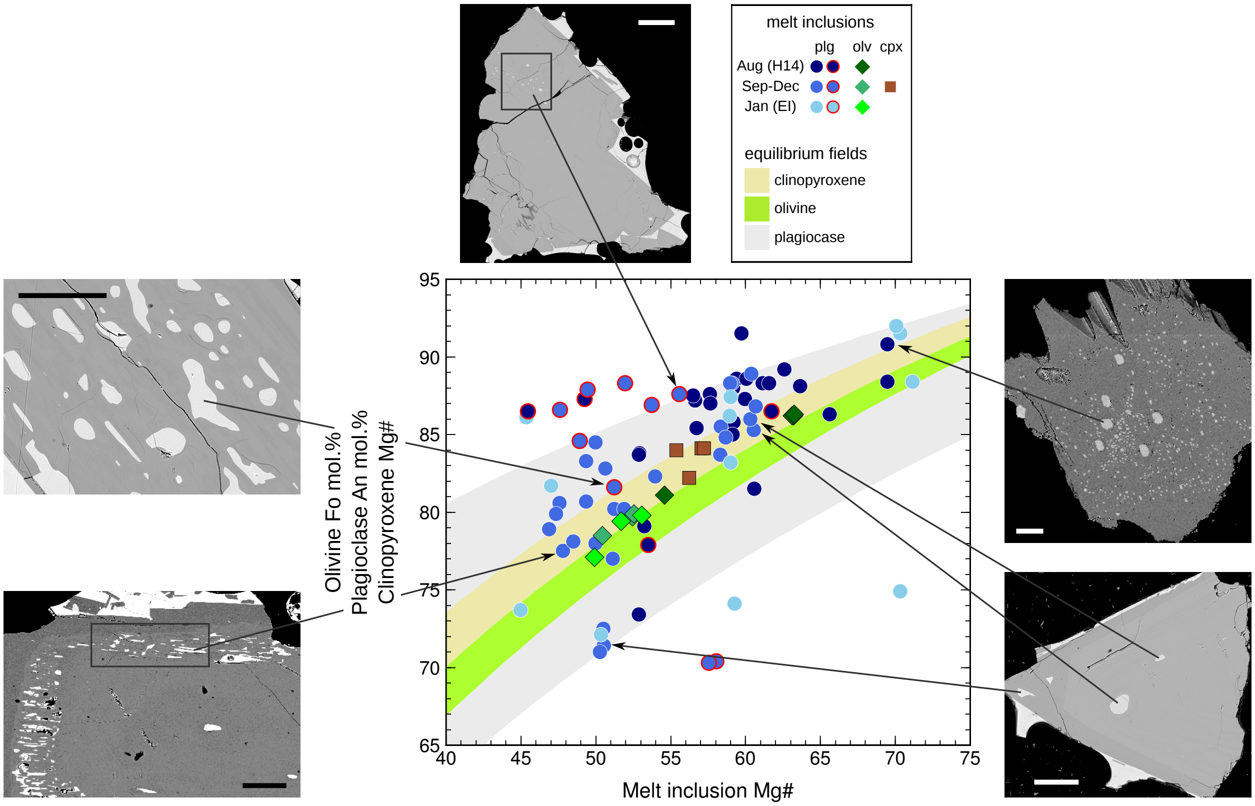

Figure S3: Back-scattered electron images of typical melt inclusion textures and corresponding PEC-corrected compositions, using the same symbols as Fig. S3. The scale bar in each image is 100 μm. The equilibrium compositional fields show Kd^{ol-liq}_{Fe-Mg}=0.30±0.015 and $Kd^{cpx-liq}_{Fe-Mg}$=0.27±0.015, and $Kd^{plg-liq}_{An-Ab}$=0.27±0.11 (Putirka, 2008). Most secondary and pseudosecondary inclusions, particularly those lying along healed fractures, were too small to analyse by SIMS or EPMA.

3. Compositional variation in Holuhraun melt inclusions

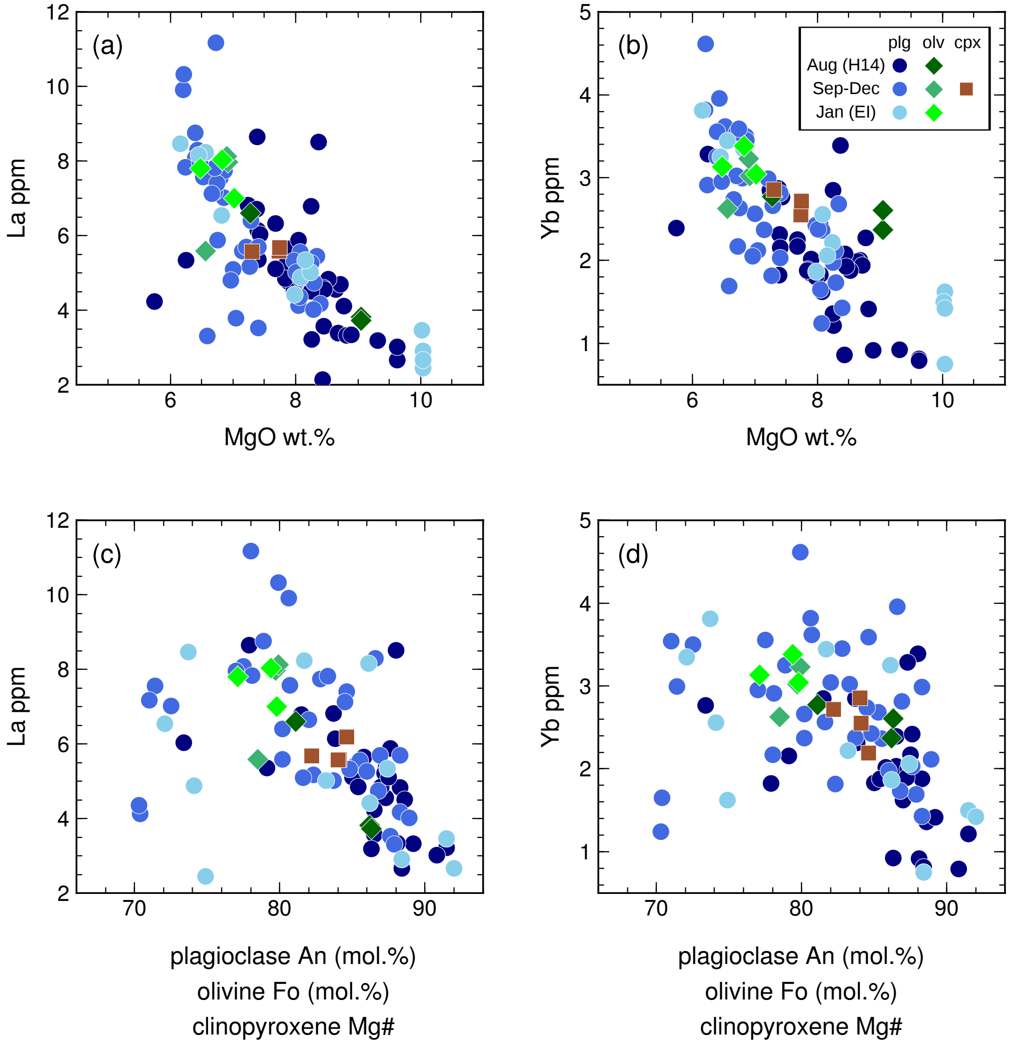

Figure S4: Variation in incompatible trace element contents in melt inclusions. (a-b) La and Yb are negatively correlated with PEC-corrected melt inclusion MgO. (c-d) La and Yb show a general negative correlation with host macrocryst composition, although there is some variability in the data. The observed trends are consistent with a fractional crystallization control on melt inclusion compositions.

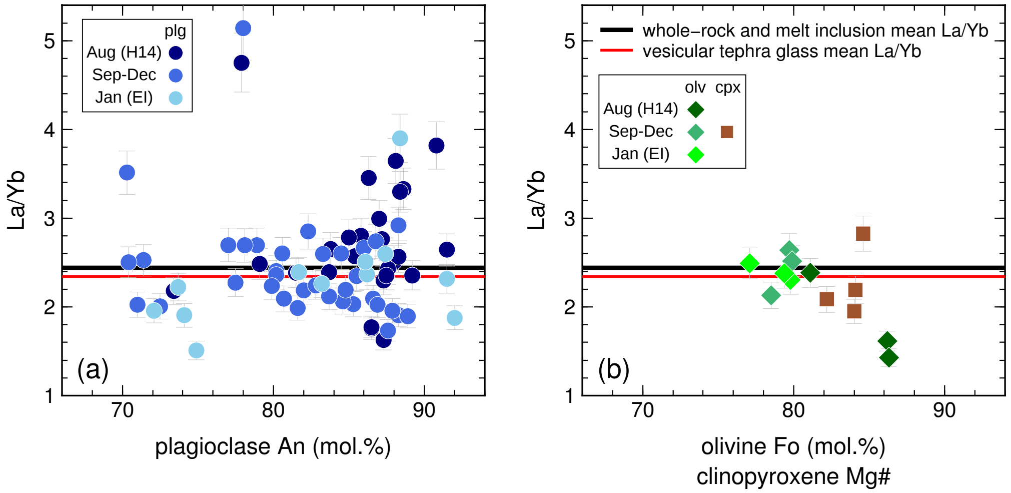

Figure S5: La/Yb vs. host macrocryst composition for Holuhraun melt inclusions.

4. Temporal evolution of melt inclusion compositions

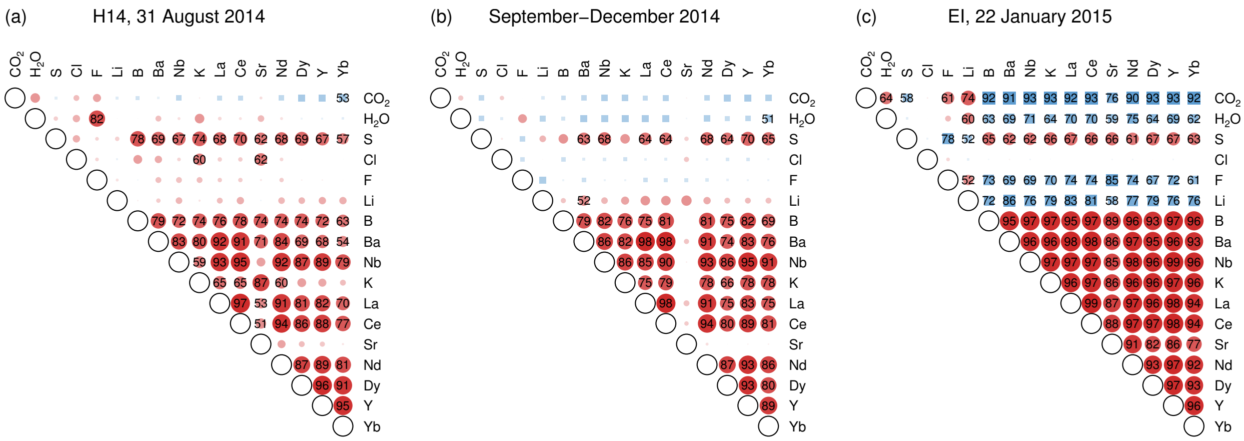

Figure S6: Correlation matrices for PEC-corrected Holuhraun melt inclusions in samples collected at different stages of the eruption. Dates of sample collection are indicated above each plot. The correlation coefficients for sample EI are high due to the wide range of melt inclusion compositions (MgO between 6.2 and 10.0 wt.%) present.

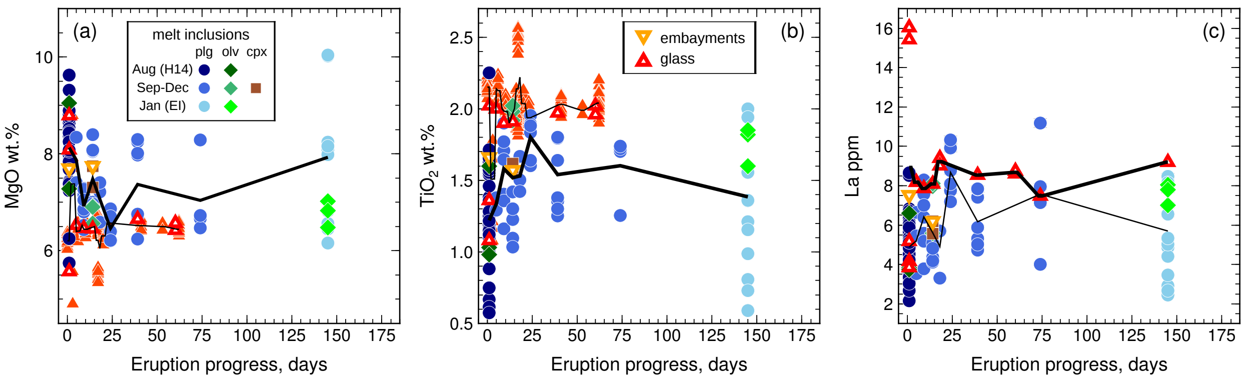

Figure S7: Variation in melt inclusion and glass compositions over the course of the eruption, with samples plotted according to their date of collection. In each plot the thick black line shows the running average melt inclusion composition calculated using a boxcar filter with bandwidth of 1 day; the thin black line shows the running average glass composition.

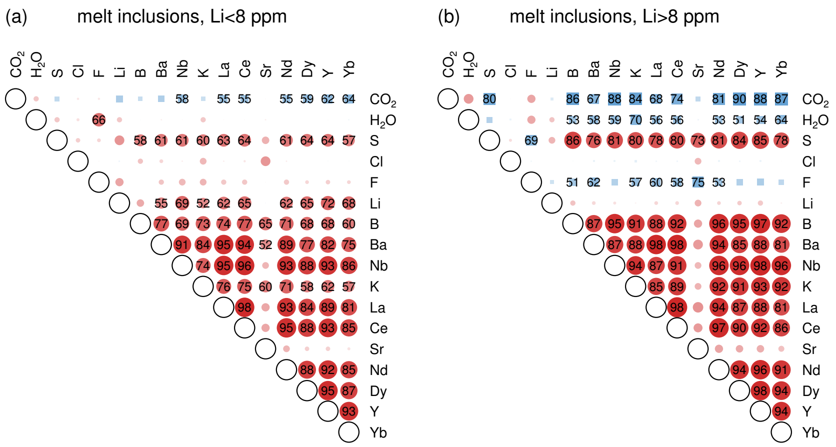

Figure S8: Correlation matrices for the low- and high-lithium populations of Holuhraun melt inclusions. (a) For melt inclusions with Li<8 ppm, Li correlates positively with incompatible trace elements but shows no significant correlation with any volatile species. (b) For the high-Li melt inclusion population, there is no correlation between Li concentration and any other incompatible element or volatile species.

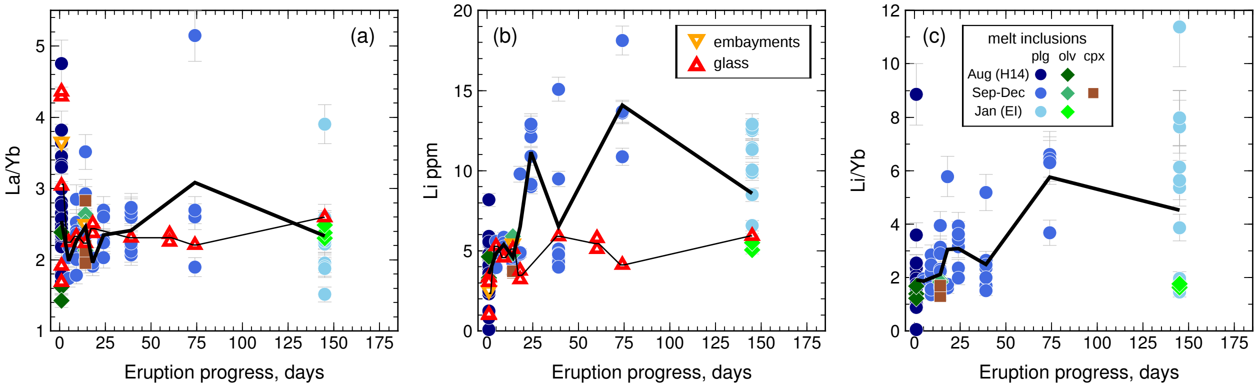

Figure S9: Variation in melt inclusion and glass compositions over the course of the eruption, using same symbols as Fig. S8. (a) Variability in melt inclusion La/Yb decreases over first 60 days of the eruption, and subsequently increases. There is no significant time-dependent variability in the mean melt inclusion or glass La/Yb. (b) Melt inclusions with high Li concentrations >8 ppm are found throughout the eruption. For the high-Li inclusions there is a weak positive correlation between eruption progress and Li content over the first 75 days of the eruption (R^2=0.34), but this correlation does not persist over the whole course of the eruption. (c) After the first day of the Holuhraun eruption, variability in Li/Yb and maximum Li/Yb both increase continuously with eruption progress. There is some indication that the mean melt inclusion Li/Yb increases over the course of the eruption.

5. Olivine-Plagioclase-Augite-Melt (OPAM) barometry

Our implementation of the Yang et al. (1996) parametrisation was initially tested on a suite of experiments conducted on a multiply saturated basalt from the Reykjanes Ridge. These tests returned good fits to the χ^2 fits with probabilities of fit P_F>0.9; however, the returned pressures were significantly greater than the true experimental pressures, with a systematic overestimate of 1.4±0.4 kbar. Similarly, a multiply saturated low-H_2O MORB composition equilibrated at an experimental pressure of 2.0 kbar (run 127 from Berndt et al. (2005)) returned an OPAM pressure of 4.5 kbar and a poor probability of fit (χ^2=31, P_F<0.1). Experiments on Shatsky Rise samples conducted at pressures of 1-7 kbar (Husen et al., 2016) had generally poor χ^2 fits, with only one of 41 experiments returning P_F>0.9. For these data the systematic pressure overestimate was 1.86 kbar, and for each defined experimental pressure there is significant variability in the returned OPAM pressure (typically 1σ=2 kbar). However, the Shatsky Rise experiments tend to be for more alkaline and more hydrous compositions than either MORB or Holuhraun melt inclusions and glasses. If only the 19 almost-dry samples in the Shatsky Rise dataset are considered then the systematic pressure overestimate drops to 0.41 kbar, although with random error of 1.4 kbar.

In order to obtain a statistically rigorous implementation of the Yang et al. (1996) OPAM barometer we tested our method against 157 experiments with multiple saturation (Fig. S8a). Across this full dataset, our implementation of the Yang et al. (1996) parametrisation provides a systematic pressure overestimate of 1.0 kbar, with a substantial random error of 2.5 kbar (Fig. S8b). When the error in the pressure estimate is plotted as a function of the probability of fit P_F according to the χ^2 estimate (Fig. S8c), a clear pattern emerges. About 60% of the experiments provide a probability of less than 0.8, where the ability of the Yang et al model to simultaneously fit X_{Mg}, X_{Ca} and X_{Al} is poor. For the 40% of experiments with a probability of over 0.8, we find a systematic pressure overestimate of 0.34 kbar with respect to the true experimental pressures, and random error of 1.32 kbar (Fig. S8d). Of the 67 experiments with $P_F \geq 0.8$, 41 are from the Yang et al. (1996) calibration dataset; 22 from Thy et al. (2006); 2 from Whitaker et al. (2007) and one from each of Toplis and Carroll (1995) and Husen et al. (2016).

An example Python script using our implementation of the OPAM barometer is available from the authors on request.

5.1 Comparison with Kelley and Barton (2008)

Here we compare the results of our implementation of the Yang et al. (1996) OPAM barometer with the widely used EXCEL-based approach of Kelley and Barton (2008) (henceforth KB08). The results of the two methodologies were compared using the original experimental dataset (n=41) used by Yang et al. (1996) to calibrate the OPAM barometer (Fig. S9). Our approach precisely reproduces the experimental data with r^2=0.93 and a standard deviation of ±1.1 kbar. In contrast, the KB08 implementation returns a poorer fit to the experimental

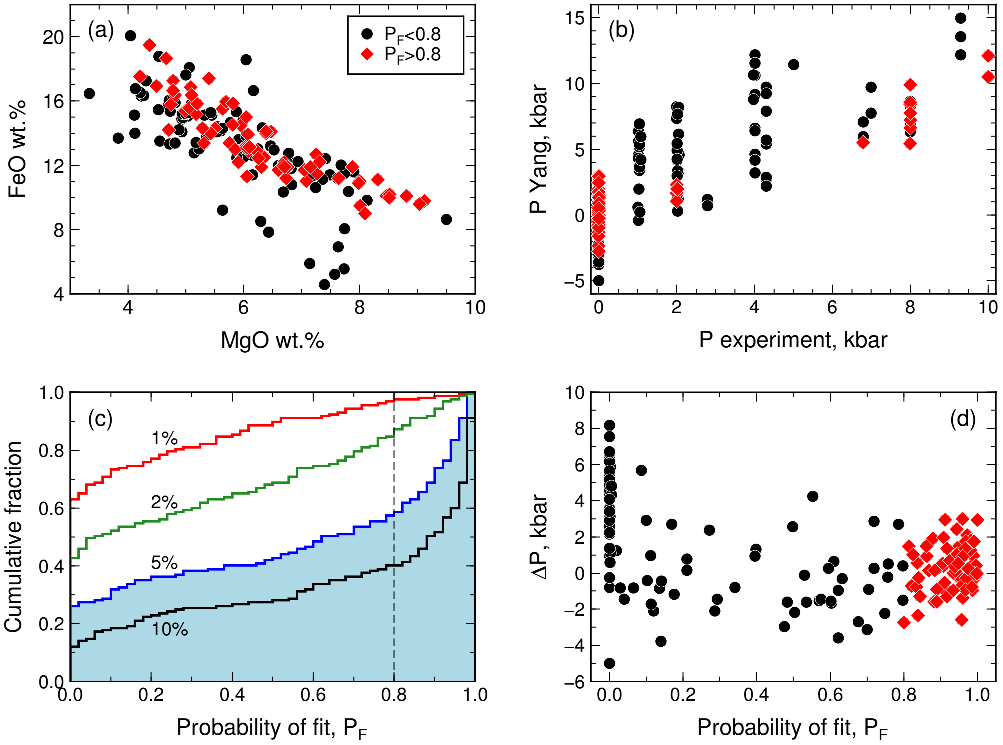

Figure S10: (a) Compositions of liquids from experiments multiply saturated in olivine, plagioclase and clinopyroxene used to test the application of the Yang et al. (1996) parametrisation. (b) Comparison of experimental pressures with those calculated using the Yang et al. (1996) OPAM barometer. In both plots, red diamonds show experiments where the returned probability of fit P_F is greater than 0.8. (c) Cumulative histogram of probability of fit P_F for experimental glasses that are reported to be multiply saturated in olivine, plagioclase and clinopyroxene. Coloured lines refer to the combined uncertainty assumed for the major element oxide components. The lower the uncertainty, the fewer melt compositions pass the P_F>0.8 filter. (b) Relationship between error in barometry (i.e. predicted pressure minus experimental pressure) and probability of fit.

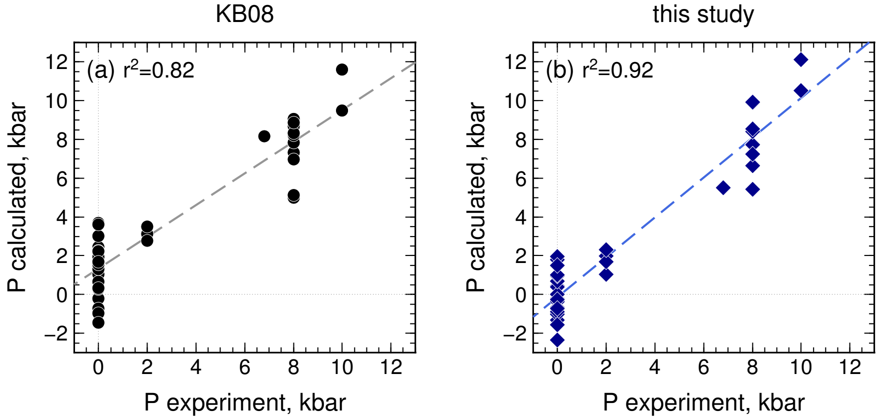

Figure S11: Comparison of OPAM barometry calculations by the model of Kelley and Barton (2008) (KB08) and this study, for the experimental compositions used to calibrate the Yang et al. (1996) paramterisation.

data (r^2=0.85). When pressures calculated using the Kelley and Barton (2008) spreadsheet are plotted against the true experimental pressures, a 1:1 correlation is not obtained. Calculated pressures for the 1 atmosphere experiments are widely scattered, with the KB08 implementation overestimating these pressures by an average 1.34 kbar (Fig. S9)

Further comparative tests were conducted using an experimental dataset expanded to include a number of experimental datasets published since 1996 which have produced olivine, plagioclase and clinopyroxene in equilibrium with melt compositions similar to Icelandic tholeiitic basalts. Using this extended dataset (n=52), our methodology again reproduces the experimental data with good precision and accuracy with a small standard deviation of ±1.1 kbar), while the KB08 spreadsheet again returns widely scattered pressures for the 1 atmosphere experiments (Fig. S10a).

A close inspection of the output from KB08 indicates that, in those cases where the pressure range (‘P-range’ - column W in the results worksheet of KB08) is greater than 2 kbar, the returned pressures are not reliable (Fig. S10b). If these estimates are omitted, the KB08 approach still seems to overestimate the true experimental pressures by an average ∼0.9 kbar (1σ=1.3 kbar). This indicates that OPAM pressures obtained by the KB08 approach should be used with caution. To minimise uncertainty, we recommend that the following be taken into consideration when processing results from the KB08 worksheet:

1. All results with ‘P-range’ >2kbar should be discarded;
2. For any remaining results where ‘P-range’ <2kbar, there may be a pressure overestimation of ∼0.8-1 kbar, particularly if the returned pressure is <2 kbar.

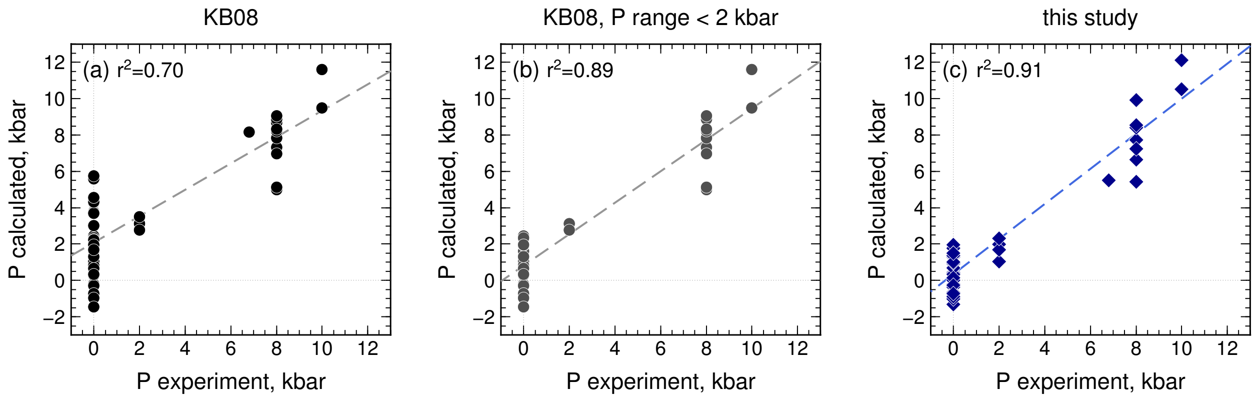

Figure S12: Comparison of OPAM barometry calculations by the model of Kelley and Barton (2008) (KB08) and this study, for experiments from an extended calibration dataset. Panels (a) and (c) show the results of all melt compositions in this dataset, while panel (b) shows only those compositions for which the 'P-range' returned by KB08 is less than 2 kbar.

5.2 Pressures of melt inclusion trapping

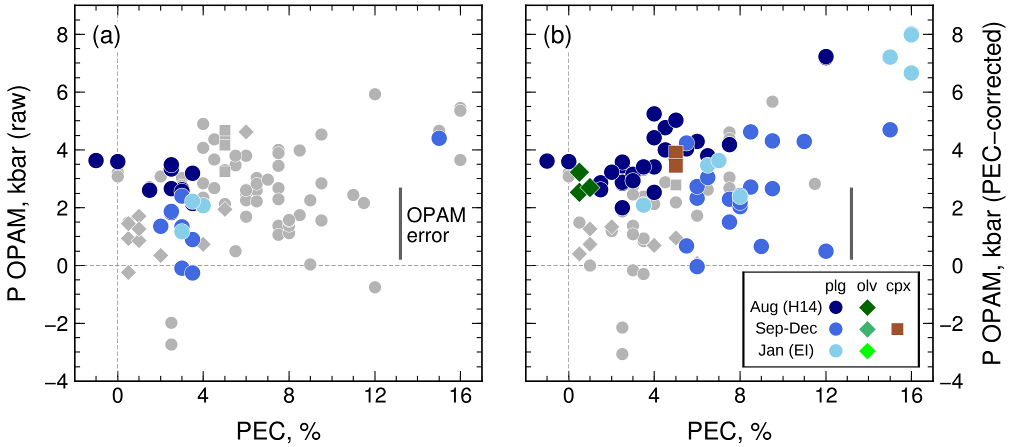

Figure S13: Melt inclusion olivine-plagioclase-augite-melt (OPAM) equilibration pressures calculated using the Yang et al. (1996) parametrisation versus extent of post-entrapment crystallization. Coloured symbols show inclusions with acceptable fits ($P_F \geq 0.8$) to the χ^2 distribution of returned pressures; grey symbols show melt inclusions where $P_F < 0.8$. (a) OPAM equilibration pressures calculated using raw melt inclusion compositions. (b) OPAM equilibration pressures calculated using PEC-corrected compositions. The maximum returned equilibration pressure increases systematically with the extent of PEC experienced by the melt inclusions; however, there is no statistically significant correlation between equilibration pressure and PEC (R^2=0.24).

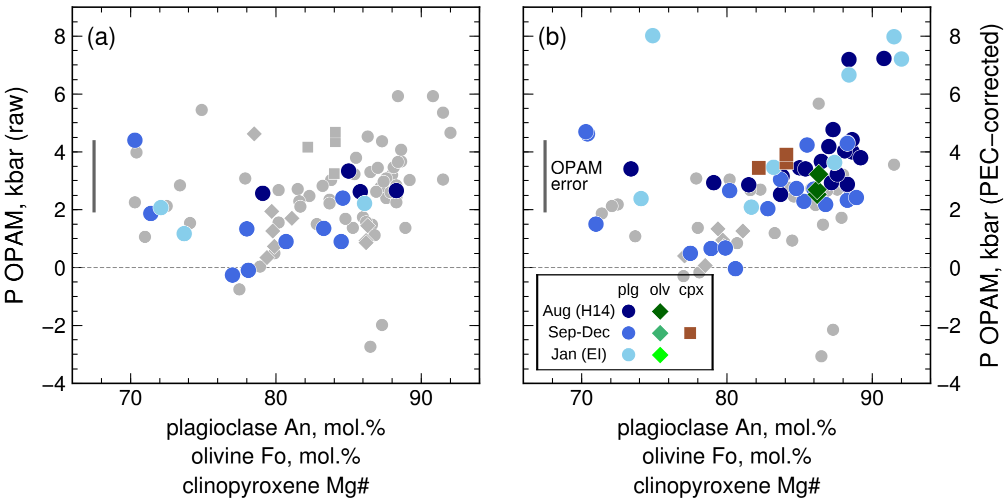

Figure S14: Melt inclusion OPAM equilibration pressures versus host macrocryst composition. Coloured symbols show inclusions with acceptable fits ($P_F \geq 0.8$) to the χ^2 distribution of returned pressures; grey symbols show melt inclusions where $P_F < 0.8$. (a) OPAM equilibration pressures calculated using raw melt inclusion compositions. (b) OPAM equilibration pressures calculated using PEC-corrected compositions. There is no statistically significant correlation between the returned equilibration pressure and the extent of PEC experienced by the melt inclusions. In both plots there is an apparent positive correlation between melt inclusion equilibration pressure and host macrocryst composition. For host plagioclase compositions more primitive than An_{75}, these correlations have R^2 values of 0.51 (raw) and 0.55 (PEC-corrected); however, if the full range of host plagioclase compositions is taken into account then the correlations are not statistically significant ($R^2 < 0.15$).

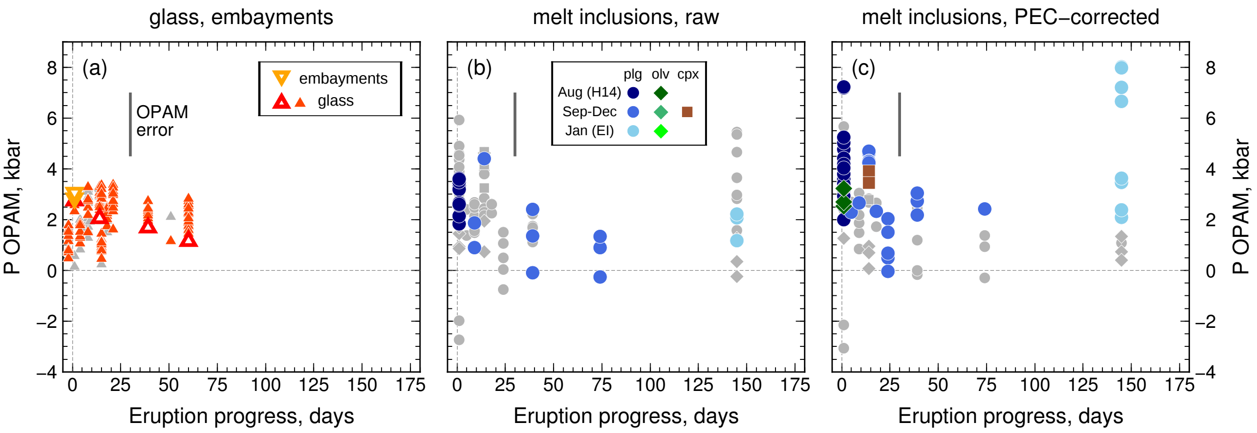

Figure S15: Variation in melt inclusion OPAM equilibration pressures over the course of the eruption, with samples plotted according to their date of collection. Coloured symbols show inclusions with acceptable fits ($P_F \geq 0.8$) to the χ^2 distribution of returned pressures; grey symbols show melt inclusions where $P_F < 0.8$. (a) OPAM equilibration pressures calculated using raw melt inclusion compositions. (b) OPAM equilibration pressures calculated using PEC-corrected compositions. With the exception of sample EI collected on day 145 of the eruption, there is an indication that melt inclusions were trapped at progressively shallower levels within the Holuhraun magmatic plumbing system. This may reflect changing depths of crystal entrainment over the course of the eruption.

References

Armstrong, J. T., 1991: Quantitative elemental analysis of individual microparticles with electron beam instruments. *Electron Probe Quantitation*, K. F. J. Heinrich and D. E. Newbury, eds., Plenum Press, 261–315.

Berndt, J., J. Koepke, and F. Holtz, 2005: An experimental investigation of the influence of water and oxygen fugacity on differentiation of MORB at 200 MPa. *Journal of Petrology*, **46**, 135–167.

Danyushevsky, L. V. and P. Plechov, 2011: Petrolog3: Integrated software for modeling crystallization processes. *Geochemistry Geophysics Geosystems*, **12**, 1–32, doi:10.1029/2011GC003516.

Hartley, M. E., J. Maclennan, M. Edmonds, and T. Thordarson, 2014: Reconstructing the deep CO_2 degassing behaviour of large basaltic fissure eruptions. *Earth and Planetary Science Letters*, **393**, 120–131.

Husen, A., R. R. Almeev, and F. Holtz, 2016: The effect of H_2O and pressure on multiple saturation and liquid lines of descent in basalt from the Shatsky Rise. *Journal of Petrology*, **57**, 309–344.

Kelley, D. F. and M. Barton, 2008: Pressures of Crystallization of Icelandic magmas. *Journal of Petrology*, **49**, 465–492.

Putirka, K., 1999: Clinopyroxene plus liquid equilibria to 100 kbar and 2450 K. *Contributions to Mineralogy and Petrology*, **135**, 151–163.

Putirka, K. D., 2008: Thermometers and barometers for volcanic systems. *Reviews in Mineralogy and Geochemistry*, **69**, 61–120.

Roedder, P. L., 1984: Fluid inclusions. *Reviews in Mineralogy*, **12**, 1–646.

Roeder, P. L. and R. F. Emslie, 1970: Olivine-liquid equilibrium. *Contributions to Mineralogy and Petrology*, **29**, 275–289.

Thy, P., C. E. Lesher, T. F. D. Nielsen, and C. K. Brooks, 2006: Experimental constraints on the Skaergaard liquid line of descent. *Lithos*, **92**, 154–180.

Toplis, M. J. and M. R. Carroll, 1995: An experimental study of the influence of oxygen fugacity on Fe-Ti oxide stability, phase relations, and mineral-melt equilibria in ferrobasaltic systems. *Journal of Petrology*, **36**, 1137–1170.

Whitaker, M. L., H. Nekvasil, D. H. Lindsley, and N. J. Difrancesco, 2007: The role of pressure in producing compositional diversity in intraplate basaltic magmas. *Journal of Petrology*, **48**, 365–393.

Yang, H.-J., R. J. Kinzler, and T. L. Grove, 1996: Experiments and models of anhydrous, basaltic olivine-plagioclase-augite saturated melts from 0.001 to 10 kbar. *Contributions to Mineralogy and Petrology*, **124**, 1–18.
